# CEBPA repression by MECOM blocks differentiation to drive aggressive leukemias^∗^

**DOI:** 10.1182/blood.2025028954

**Published:** 2025-09-26

**Authors:** Travis J. Fleming, Mateusz Antoszewski, Sander Lambo, Michael C. Gundry, Riccardo Piussi, Lara Wahlster, Sanjana Shah, Fiona E. Reed, Kevin D. Dong, Joao A. Paulo, Steven P. Gygi, Claudia Mimoso, Seth R. Goldman, Karen Adelman, Jennifer A. Perry, Yana Pikman, Kimberly Stegmaier, Maria N. Barrachina, Kellie R. Machlus, Volker Hovestadt, Andrea Arruda, Mark D. Minden, Richard A. Voit, Vijay G. Sankaran

**Affiliations:** 1 Division of Hematology/Oncology, Boston Children’s Hospital, Harvard Medical School, Boston, MA; 2 Department of Pediatric Oncology, Dana-Farber Cancer Institute, Harvard Medical School, Boston, MA; 3 Howard Hughes Medical Institute, Boston, MA; 4 Broad Institute of MIT and Harvard, Cambridge, MA; 5 Department of Cell Biology, Harvard Medical School, Boston, MA; 6 Department of Biological Chemistry and Molecular Pharmacology, Harvard Medical School, Boston, MA; 7 Vascular Biology Program, Boston Children’s Hospital, Boston, MA; 8 Department of Surgery, Boston Children’s Hospital, Harvard Medical School, Boston, MA; 9 Princess Margaret Cancer Centre, University Health Network, Toronto, ON, Canada; 10 Department of Medical Biophysics, University of Toronto, Toronto, ON, Canada; 11 Harvard Stem Cell Institute, Cambridge, MA

## Abstract

Acute myeloid leukemias (AMLs) have an overall poor prognosis with many high-risk cases co-opting stem cell gene regulatory programs, but the mechanisms through which these programs are propogated remain poorly understood. The increased expression of the stem cell transcription factor, MECOM, underlies a key driver mechanism in largely incurable AMLs. However, how MECOM results in such aggressive AML phenotypes remains unknown. To address existing experimental limitations, we engineered and applied targeted protein degradation with functional genomic readouts to demonstrate that MECOM promotes malignant stem cell–like states by directly repressing prodifferentiation gene regulatory programs. Remarkably and unexpectedly, a single node in this network, a MECOM-bound cis-regulatory element located 42 kilobase (kb) downstream of the myeloid differentiation regulator *CEBPA* is both necessary and sufficient for maintaining MECOM-driven leukemias. Importantly, the targeted activation of this regulatory element promotes differentiation of these aggressive AMLs and reduces leukemia burden in vivo. These findings suggest a broadly applicable approach for functionally dissecting oncogenic gene regulatory networks to inform improved therapeutic strategies.

## Key Points

• MECOM promotes malignant stem cell–like states in aggressive AMLs by directly repressing prodifferentiation gene regulatory programs.
• A MECOM-bound cis-regulatory element 42 kb downstream of CEBPA sustains AML and activating it induces differentiation.

## Visual Abstract

**Figure:**
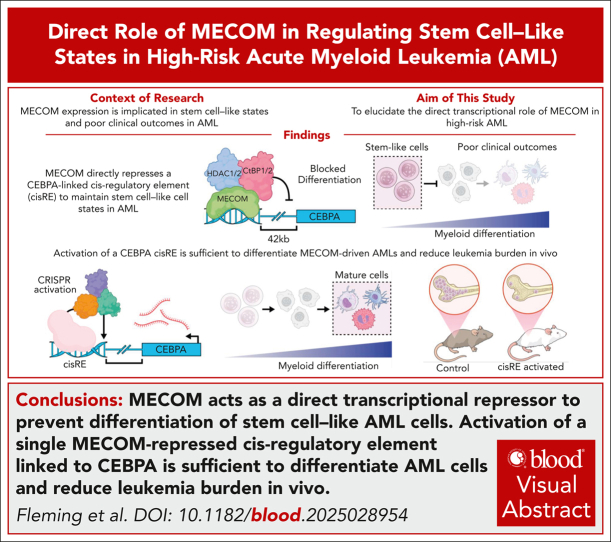

## Teaser

High-risk acute myeloid leukemia (AML) frequently exhibits increased expression of the transcription factor MDS1 and EVI1 complex locus protein (MECOM), which is essential for hematopoietic stem cells in healthy states. Two independent studies in this issue, a Plenary Paper by Fleming et al and a Brief Report by Pastoors et al, describe a simple mechanism whereby the MECOM protein preserves the leukemic state by binding the lineage-restricted +42 kb enhancer of the CCAAT enhancer-binding protein α (*CEBPA*) gene. Importantly, removing that repression through MECOM degradation rapidly restores *CEBPA* expression, triggering terminal myeloid differentiation. These insights lay the groundwork for potential new differentiation therapies for high-risk AML.

## Introduction

Acute myeloid leukemia (AML) is an aggressive blood cancer with a cure rate below 30%,^1^ largely reflecting disease heterogeneity and resistance to standard chemotherapy.^2^^,^^3^ Beyond genetic drivers, the persistence of hematopoietic stem cell (HSC) gene expression programs contributes to AML aggressiveness and relapse.^4^, ^5^, ^6^, ^7^ Although therapies such as venetoclax^8^ and menin inhibitors^9^ target leukemia stem cells, few restore differentiation programs,^9^ as shown in acute promyelocytic leukemia (APL).^10^

High-risk AMLs frequently exhibit increased MECOM expression, sustaining stem cell–like states.^11^^,^^12^ Although conventional loss-of-function studies have started dissecting MECOM’s direct role, the interpretation of direction function can be confounded by cell state changes.^13^, ^14^, ^15^, ^16^, ^17^, ^18^, ^19^, ^20^ To overcome this, we applied targeted protein degradation and functional genomic assays, showing MECOM enforces stem cell–like features by repressing a single *CEBPA* cis-regulatory element (cisRE). The transient activation of this element induces differentiation and reduces leukemia burden in vivo. These findings support differentiation-based therapies in high-risk AMLs.

## Methods

### Overview

Human AML cell lines and primary AML blasts were cultured in defined media and engineered via CRISPR/CRISPR-associated protein 9 (Cas9) genome editing either to tag *MECOM* with an FKBP12^F36V^ degron or to edit *MECOM* or a *CEBPA* cisRE. MECOM degradation was triggered with dTAG^V^-1, and effects were evaluated by RNA sequencing (RNA-seq), assay for transposase-accessible chromatin with high-throughput sequencing (ATAC-seq), precision run-on sequencing (PRO-seq), and tandem mass tag proteomics. Leukemia-initiating potential was assessed by xenotransplantation into NOD.Cg-*Kit*^W-41J^*Tyr*^+^*Prkdc*^scid^*Il2rg*^tm1Wjl^ (NBSGW) mice, with engraftment and differentiation analyzed by flow cytometry, histology, and molecular expression analyses.

### Statistics

Statistical tests and significance thresholds are provided in figure legends. Error bars represent standard error of the mean unless noted. RNA-seq, ATAC-seq, chromatin immunoprecipitation sequencing (ChIP-seq), and PRO-seq were analyzed using DESeq2, MACS2, and deepTools (see supplemental Methods, available on the *Blood* website). Gene ontology and enrichment analyses used Genome Regions Enrichment of Annotations Tool (GREAT), gene set enrichment analysis (GSEA) package in python (GSEApy), and GSVA. CRISPR screen analysis used MAGeCKFlute. Pearson/Spearman correlations, Wilcoxon, *t* tests, and Mann-Whitney tests were applied as indicated.

### Patient material

Primary AML samples were collected with informed consent and approved by ethics boards at Boston Children’s Hospital/Dana-Farber or the University Health Network. Mononuclear cells were isolated from bone marrow or blood and cryopreserved. Xenotransplant procedures were approved by the Institutional Animal Care and Use Committee at Boston Children’s Hospital.

All other methods are described in detail in the supplemental Methods.

## Results

### Rapid and specific protein degradation enables direct interrogation of MECOM function in AML

**Figure 1.**
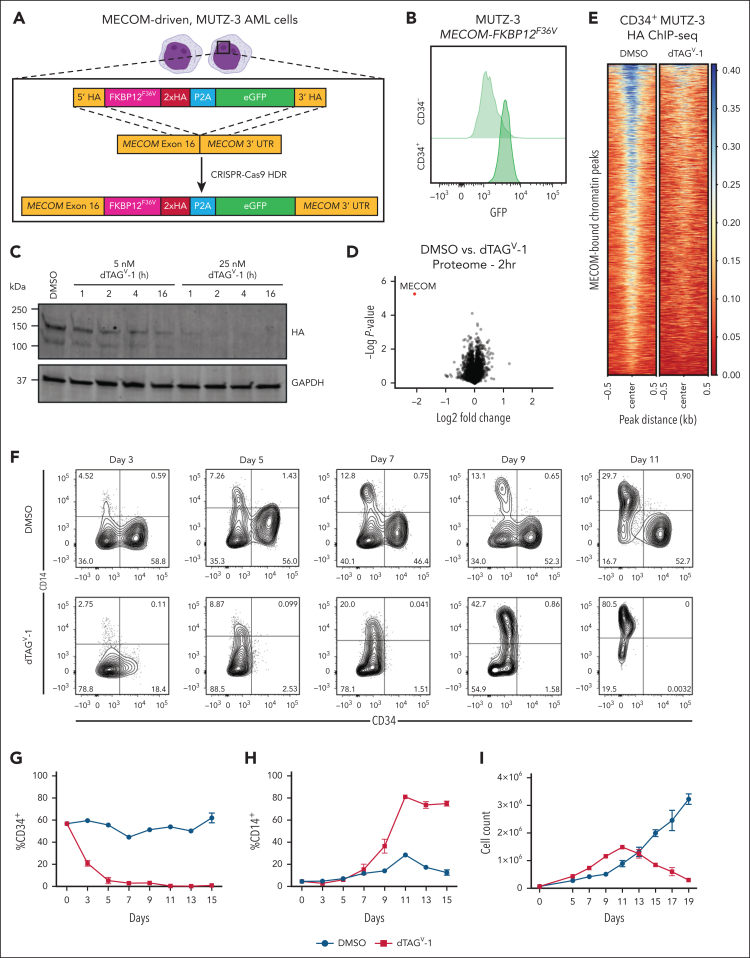
**FKBP12^F36V^ degron facilitates rapid degradation of endogenous MECOM in AML cells.** (A) Schematic illustrating the gene-editing strategy to knock in an FKBP12^F36V^ degron, 2xHA tag, and eGFP at the C-terminus of the endogenous *MECOM* locus in human MUTZ-3 AML cells. (B) GFP expression assessed by flow cytometry in CD34^+^ vs CD34^–^ MUTZ-3 MECOM-FKBP12^F36V^ cells. (C) Time course western blot analysis of MECOM protein levels in MUTZ-3 cells following treatment with dTAG^V^-1 (5-25 nM) or dimethyl sulfoxide (DMSO). (D) Volcano plot showing changes in protein abundance in MUTZ-3 MECOM-FKBP12^F36V^ cells treated for 2 hours with 500 nM dTAG^V^-1 vs DMSO as assessed by mass spectrometry. n = 3 independent replicates. (E) MECOM ChIP-seq of MUTZ-3 MECOM-FKBP12^F36V^ cells treated with 500 nM dTAG^V^-1 vs DMSO (n = 3). Each row represents a single MECOM(HA)-bound peak. Heat map is centered on ChIP-peak summits ±500 bp. (F) Bivariate plot showing CD34 and CD14 expression levels in MUTZ-3 MECOM-FKBP12^F36V^ cells treated with 500 nM dTAG^V^-1 vs DMSO. (G-H) Percentage of CD34^+^ and CD14^+^ cells as observed in panel F. n = 3 independent replicates. Mean and standard error of the mean (SEM) are shown. (I) Viable cell count by trypan blue exclusion of MUTZ-3 MECOM-FKBP12^F36V^ cells treated with 500 nM dTAG^V^-1 vs DMSO. n = 3 independent replicates. Mean and SEM are shown. eGFP, enhanced green fluorescent protein; GAPDH, glyceraldehyde-3-phosphate dehydrogenase; kDA, kilodalton.

To directly elucidate MECOM-driven transcriptional and epigenetic programs in stem cell–like leukemia cells, we engineered 3 AML cell line models with a 2xHA-FKBP12^F36V^-P2A-enhanced green fluorescent protein (eGFP) cassette at the C-terminus of the endogenous *MECOM* locus (Figure 1A). The synthetic FKBP12^F36V^ degron enables rapid degradation of tagged proteins with the addition of degradation tag (dTAG) small molecules.^21^^,^^22^ We selected the AML cell lines MUTZ-3, UCSD-AML1, and HNT-34 cells, considering their high *MECOM* expression level and cytogenetic status that arises due to an oncogenic translocation/inversion event that juxtaposes an enhancer of *GATA2* to drive high-level *MECOM* expression.^23^^,^^24^ Consistent with *MECOM* expression being restricted to stem cell–like populations,^12^ the GFP^+^ expression is enriched in the CD34^+^ compartment (Figure 1B). The treatment of MUTZ-3 *MECOM-FKBP12*^*F36V*^ (MUTZ3-dTAG), UCSD-AML1-dTAG, and HNT-34-dTAG cells with low nanomolar (5-500 nM) concentrations of dTAG^V^-1 resulted in the rapid degradation of all MECOM protein within 1 hour of treatment compared with that in dimethyl sulfoxide vehicle controls (Figure 1C; supplemental Figure 1A). Moreover, multiplexed quantitative mass spectrometry demonstrated that MECOM was the only protein whose abundance was significantly altered in the proteome of MUTZ-3 cells following the addition of dTAG^V^-1 for 2 hours (fold change less than –1.0; *P* < .001) (Figure 1D). To further corroborate the specificity of this approach in rapidly ablating MECOM, we measured MECOM chromatin occupancy in CD34^+^ MUTZ-3 progenitor cells treated with dTAG^V^-1, and MECOM binding was nearly lost genome-wide (Figure 1E). We next sought to validate the utility of these degron models to glean insights into the regulation of stem cell gene regulatory programs. Consistent with studies that genetically perturb *MECOM*,^12^^,^^17^^,^^18^^,^^25^ MUTZ-3-dTAG cells treated with dTAG^V^-1 exhibited nearly complete loss of CD34 expression followed by acquisition of CD14 expression, consistent with monocytic differentiation (Figure 1F-H). Although dTAG^V^-1–treated MUTZ-3 cells initially proliferate more, they eventually all die in culture presumably due to loss of stem cell/progenitor populations and the short persistence of terminally differentiated cells^12^^,^^26^ (Figure 1I). This robust myeloid differentiation phenotype was conserved in UCSD-AML1-dTAG cells, where dTAG^V^-1 treatment resulted in the loss of CD34 expression (supplemental Figure 1B-C). We did not observe signs of morphologic or immunophenotypic differentiation in HNT-34-dTAG cells following MECOM degradation; however, the cells rapidly underwent apoptosis in culture (supplemental Figure 1D-E). This result is in agreement with a previous report describing a strong MECOM dependency in HNT-34 cells.^17^ To further profile the impact of synchronous loss of MECOM, we used a fluorescent EdU-labeling assay to analyze cell cycle differences induced upon MECOM loss. dTAG^V^-1 treatment conferred a significant increase in actively dividing cells in S and G2 phases and a significant decrease in cells in G0/G1 phase (supplemental Figure 1F-G). This finding is consistent with the observed differentiation phenotypes, considering that loss of quiescence accompanies hematopoietic differentiation. All these experiments demonstrate the utility of the dTAG system to rapidly and specifically degrade MECOM in cellular models of leukemia. Importantly, these models enable sensitive molecular profiling following MECOM ablation and before cell state changes, which is crucial for elucidating its direct role in enabling stem cell phenotypes in high-risk AMLs.

### MECOM directly represses myeloid differentiation programs in AML

**Figure 2.**
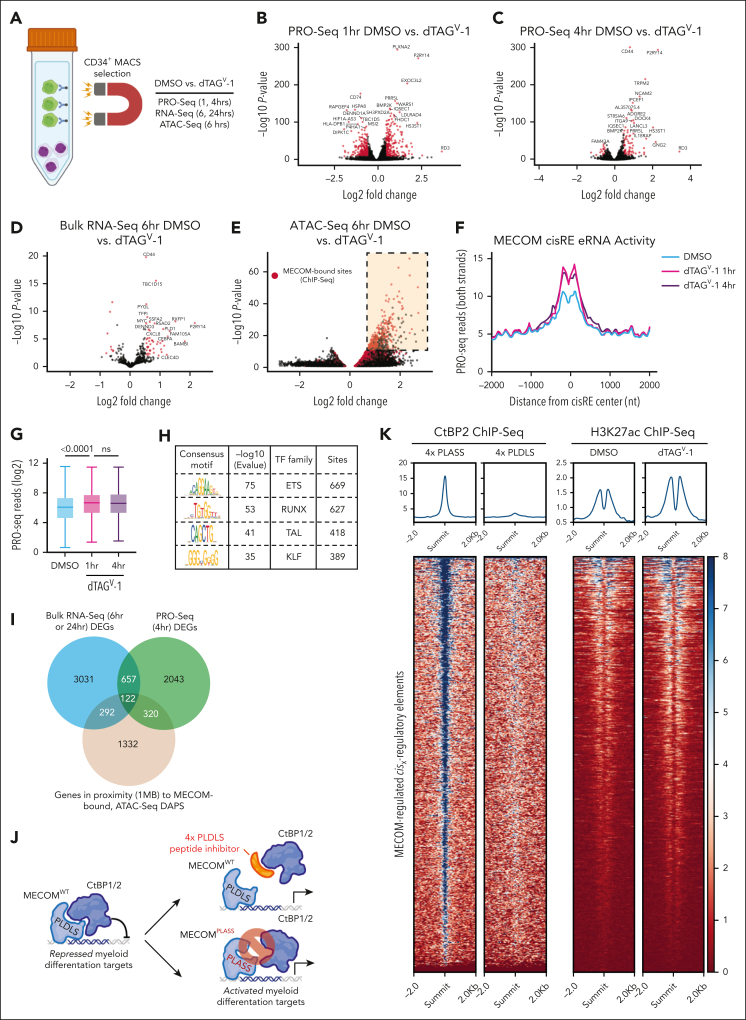

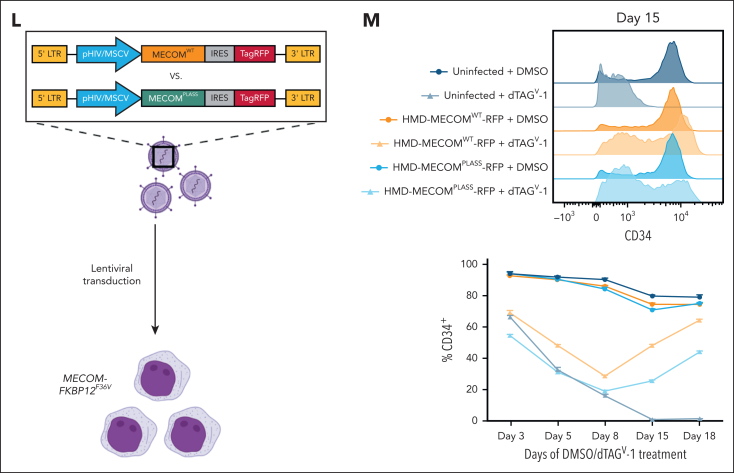
**Multiomic profiling of MECOM-depleted cells reveals a predominantly repressive role at target sites.** (A) Schematic representation of experimental protocol for multiomic characterization of dTAG^V^-1–treated MUTZ-3 MECOM-FKBP12^F36V^ cells. The CD34^+^, GFP^+^ MECOM-expressing population was preenriched via MACS before treatment with 500 nM dTAG^V^-1 or DMSO. Cells were then harvested and processed for bulk RNA-seq, ATAC-seq, and PRO-seq to profile transcriptional and epigenetic changes. (B-C) Volcano plots representing changes in nascent gene expression assessed via PRO-seq in MUTZ-3 MECOM-FKBP12^F36V^ cells treated with dTAG^V^-1 vs DMSO for 1 and 4 hours. n = 3 independent replicates (supplemental Table 4). (D) Volcano plot representing changes in gene expression assessed via bulk RNA-seq in MUTZ-3 MECOM-FKBP12^F36V^ cells treated with dTAG^V^-1 vs DMSO for 6 hours. n = 3 independent replicates (supplemental Table 3). (E) Volcano plot representing changes in chromatin accessibility as assessed by ATAC-seq in MUTZ-3 MECOM-FKBP12^F36V^ cells treated with dTAG^V^-1 vs DMSO for 6 hours. n = 3 independent replicates. Red data points represent chromatin peaks that are also bound by MECOM as assessed by MECOM-HA ChIP-seq. There are 837 of these sites that are schematically highlighted in the top right corner of the plot (supplemental Table 5). (F-G) Assessment of eRNA transcription levels at 837 MECOM-bound differentially accessible peaks measured from PRO-seq data. (F) Average PRO-seq read density across all MECOM-regulated cisREs with ±2000 bp on each side of the peak summit in dTAG^V^-1–treated vs DMSO-treated samples. (G) Box plot showing average PRO-seq read density in aggregate for each MECOM-regulated cisRE ±500 bp on each side of the peak summit in dTAG^V^-1–treated vs DMSO-treated samples. Two-sided Student *t* test was used for comparisons. n = 3 independent replicates. (H) Unbiased motif enrichment analysis of ATAC-seq differentially accessible peaks between dTAG^V^-1–treated and DMSO-treated samples. (I) Venn diagram comparing gene expression and chromatin accessibility changes across sequencing modalities. Bulk RNA-seq DEGs from 6 hours and 24 hours dTAG^V^-1 treatment, PRO-seq DEGS from 4 hours dTAG^V^-1 treatment, and genes in proximity (within 1 MB) to at least 1 MECOM-bound, differentially accessible ATAC-seq peak were overlapped to yield a consensus MECOM gene network consisting of 122 genes. Cutoffs for bulk RNA-seq and PRO-seq were *P* < .05 (supplemental Tables 6 and 7). Peak-to-gene proximity was determined using the GREAT.^31^ (J) Schematic depiction of MECOM’s interaction with transcriptional corepressor CtBP2 via MECOM’s PLDLS motif. This protein-protein interaction can be inhibited by a genetically encoded 4x-PLDLS peptide inhibitor^32^ (top) or if MECOM’s PLDLS motif were mutated to PLASS (bottom). (K) H3K27ac and CtBP2 ChIP-seq analysis. Heat map (left) displays CtBP2 ChIP-seq signal at MECOM-regulated cisREs in MUTZ-3 cells expressing a 4x-PLDLS peptide inhibitor of the MECOM-CtBP2 interaction compared with cells expressing 4x-PLASS control.^32^ Heat map (right) showing H3K27ac ChIP-seq signal at MECOM-regulated cisREs in MUTZ-3 MECOM-FKBP12^F36V^ cells treated with 500 nM dTAG^V^-1 or DMSO for 6 hours. (L-M) Experimental overview for lentiviral MECOM add-back rescue experiment. (L) MUTZ-3 MECOM-FKBP12^F36V^ cells were transduced with lentiviruses constitutively expressing either WT MECOM (EVI1 isoform) or MECOM PLDLS>PLASS along with a TagRFP transduction reporter at high MOI. (M) CD34 expression assessed by flow cytometry as a function of treatment duration (500 nM DMSO vs dTAG^V^-1) (bottom). Histogram of CD34 expression at day 15 (top). Samples were transduced 48 hours before treatment. n = 3 independent technical replicates. Mean and standard deviation are shown, but many are hidden due to low variation between replicates. DEGs, differentially expressed genes; DAPs, differentially accessible peaks; eRNA, enhancer RNA; GFP, green fluorescent protein; MACS, magnetic-activated cell sorting; MB, megabase; MOI, multiplicity of infection; ns, not significant; nt, nucleotide; TF, transcription factor; WT, wild-type.

Having established and validated several MECOM degron models, we next performed multiomic profiling following MECOM degradation to elucidate regions of accessible chromatin and genes directly regulated by this transcription factor. By restricting profiling to stem cell–like, MECOM-expressing cells, we would enhance our ability to detect direct transcriptional and epigenetic alterations. Following CD34^+^ enrichment (Figure 2A), MUTZ-3 cells were treated with 500 nM dTAG^V^-1 and analyzed for changes in nascent transcription via PRO-seq, bulk transcription (bulk RNA-seq), and chromatin accessibility (ATAC-seq). One hour after MECOM degradation, we detected both increases (468 genes, *P* < .01, Log2FoldChange [L2FC] >0.5) and decreases (600 genes, *P* < .01, L2FC < 0.5) in nascent gene expression (Figure 2B; supplemental Table 3). However, by 4 hours (PRO-seq) (Figure 2C) and 6 hours (bulk RNA-seq) (Figure 2D; supplemental Tables 3 and 4) after MECOM degradation, far more genes showed increased expression (4 hours: 153 increased vs 64 decreased genes; 6 hours: 47 increased vs 8 decreased genes, respectively), suggestive of a direct repressive function for MECOM in this context. Moreover, a recent study from our group elucidated an HSC gene signature that is downregulated upon MECOM perturbation in primary human HSCs (MECOM down genes).^12^ Notably, far more MECOM down genes show significantly reduced expression at 24 hours vs 6 hours after MECOM degradation (58 genes vs 1 gene, respectively, L2FC less than –0.5; *P* < .001; supplemental Figure 2A-D), highlighting how the loss of stem cell maintenance gene programs in AML is likely to be secondary to the activation of myeloid differentiation programs observed upon the acute loss of MECOM.

The analysis of genome-wide chromatin accessibility 6 hours after MECOM degradation showed a significant skew toward regions with increased accessibility, further corroborating this primarily repressive role for MECOM (Figure 2E; supplemental Table 5). Specifically, accessible chromatin regions (*P* < .0001) showed strong increases in accessibility (3071/3462 peaks [88.7%] with L2FC > 0.5, *P* < .0001) compared with a small number of peaks with decreased accessibility (155/3462 peaks [4.48%] with L2FC less than –0.5; *P* < .0001). Moreover, when overlapping these differentially accessible peaks with MECOM ChIP-seq peaks (highlighted in red), we observed a striking enrichment of overlapped peaks that show increases in accessibility (1602/3071 overlapping peaks [52.2%] vs 44 425/308  630 [14.4%] of ChIP-seq peaks overlapping any accessible chromatin region). We further conducted restricted analysis to a subset of these differentially accessible sites (837) with strong MECOM chromatin occupancy (ChIP-seq *P* score [–log10 *P* value∗10] > 50) and henceforth refer to this network of MECOM-bound sites as the direct MECOM cisRE network (supplemental Table 6). Across this cisRE network, we detected an increase in enhancer RNA transcription, which serves as a proxy for enhancer activity,^27^^,^^28^ following MECOM degradation (Figure 2F-G). Moreover, transcription factor motif enrichment analysis of this MECOM cisRE network revealed strong enrichment of ETS motifs (Figure 2H), which was consistent with prior reports of the binding specificity of MECOM’s C-terminal zinc finger domain.^29^^,^^30^ To define a consensus, directly regulated MECOM gene signature, we integrated results from these multiomic readouts. For this purpose, we first employed the GREAT^31^ to link our MECOM cisRE network to genes by proximity. This analysis nominated 1332 genes that were within 1 megabase (MB) of at least 1 cisRE. We then took the union of this gene set, the differentially expressed genes from bulk RNA-seq (6 hours/24 hours, *P* < .05) and those obtained from PRO-seq (4 hours, *P* < .05) to define a consensus network of 122 genes that might be under the direct regulation of MECOM (Figure 2I; supplemental Table 7). To validate that these MECOM-regulated cisRE and gene networks that were conserved across multiple AML models, we performed GSEA of these MECOM-regulated cisRE and gene networks in UCSD-AML1-dTAG and HNT-34-dTAG cells and showed a strong enrichment of both networks in cells treated with dTAG^V^-1 vs dimethyl sulfoxide via bulk RNA-seq and ATAC-seq (supplemental Figure E-L). Collectively, these results indicate that MECOM promotes stem cell–like phenotypes in AML by repressing a highly conserved myeloid differentiation program.

Given these findings, we hypothesized that MECOM’s repressive role might be enabled by its interaction with transcriptional co-repressors. Notably, MECOM has previously been shown to bind the C-terminal binding proteins 1 and 2 (CtBP1/2) through a PLDLS motif,^32^, ^33^, ^34^ and this interaction can be blocked through the addition of a peptide inhibitor^32^ (Figure 2J). We analyzed CtBP2 ChIP-seq data^32^ from MUTZ-3 cells treated with this peptide inhibitor and observed a loss of CtBP2 binding in our MECOM cisRE network, consistent with a model in which MECOM recruits CtBP2 to repress cisREs (Figure 2K). Consistent with the loss of CtBP2 occupancy, MECOM degradation also conferred a significant increase in H3K27 acetylation across the MECOM cisRE network (Figure 2K). To further validate the significance of this interaction, we performed a lentiviral rescue experiment with either exogenously expressed MECOM^WT^ or MECOM^PLDLS>PLASS^ (an isoform unable to interact with CtBP1/2) to rescue dTAG^V^-1–induced differentiation (Figure 2L). In line with the importance of this interaction for the repressive function of MECOM, MECOM^WT^ rescued the loss of CD34^+^ progenitor cells induced by dTAG^V^-1 treatment of MUTZ-3 cells to a greater extent than MECOM^PLDLS>PLASS^ (Figure 2M).

### MECOM gene regulatory networks are highly conserved in primary AMLs

**Figure 3.**
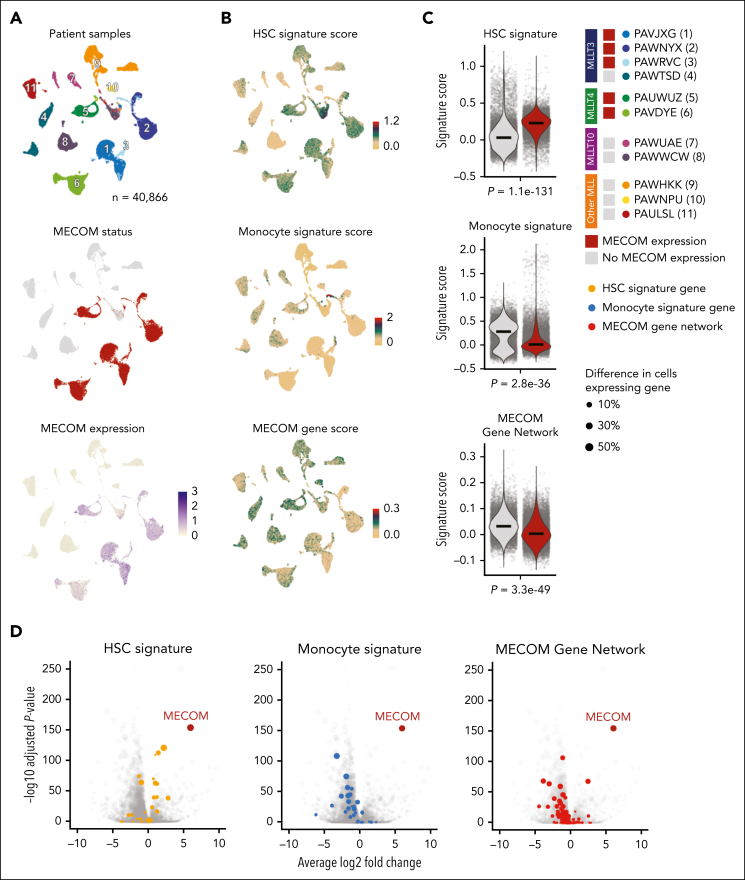
**Direct MECOM gene network is repressed in primary leukemia cells.** (A) UMAP of 40 866 cells derived from 11 patients with leukemias driven by MLLrs sequenced using scRNA-seq. UMAPs were colored from top to bottom by patient, whether MECOM is expressed, and MECOM expression counts per cell. (B) Same UMAPs as panel A and colored by the expression signatures of normal HSCs and normal monocytes derived from Lambo et al^36^ and MECOM-regulated genes identified to be activated after depletion of MECOM (Figure 2). (C) Quantification of the 3 signatures from panel B compared between MECOM-positive leukemias (n = 5) and MECOM-negative leukemias (n = 6). Comparisons were performed by randomly taking the average over 10 iterations of 1000 randomly sampled cells from both samples expressing MECOM and samples not expressing MECOM to avoid uninformative *P* values close to 0. Significance was calculated using 2-sided Wilcoxon signed-rank tests corrected for multiple testing using BH. (D) Differential expression of all analyzed genes (n = 28 113) between leukemias expressing MECOM and leukemias that did not express MECOM. Differential expression was performed using MAST using 10 iterations of 1000 randomly selected MECOM-positive cells and 1000 randomly selected MECOM-negative cells to prevent uninformative *P* values. BH corrected *P* values and log fold changes shown are the average of 10 iterations. BH, Benjamini-Hochberg; UMAP, uniform manifold approximation and projection.

We sought to examine how these genetic programs might be conserved in primary leukemias. We leveraged bulk transcriptome, single-cell RNA-seq (scRNA-seq), and single-cell ATAC-seq (scATAC-seq) from pediatric patients enrolled in the AAML1031 clinical trial^35^^,^^36^ to investigate whether gene expression and chromatin accessibility changes observed after MECOM depletion were present in patients with high *MECOM* expression compared with those without *MECOM* expression. A total of 67 of 701 AML samples had high expression of *MECOM* (log_2_ expression > 5). The majority of leukemias expressing *MECOM* were driven by MLL rearrangements (MLLr) (59.7%), with other notable rearrangements being NUP98 fusions and MECOM fusions, confirming previously described subgroup specific patterns of MECOM expression^37^ (supplemental Figure 3A). Notably, the level of *MECOM* expression in MECOM-fusion AMLs from this cohort was higher than in most other samples, except for samples with MLLT4 fusions. We focused on MLLr AMLs, given that this comprised the largest cohort (supplemental Figure 3B-C). Overall survival and event-free survival was significantly worse in patients with AML with detectable/high *MECOM* expression (supplemental Figure 3D), underscoring the importance of MECOM’s gene regulatory activity in relation to patient survival, as has been reported previously.^38^^,^^39^ Within the cohort, *MECOM* expression also correlated with significantly higher levels of HSC-associated genes CD34 and SPINK2 (supplemental Figure 3E). Similar to previous reports,^40^ *MECOM* expression correlated with a scRNA-seq–derived nonmalignant HSC signature,^36^ further supporting MECOM’s role in enabling stem cell–like gene expression programs in AML (supplemental Figure 3F-G). Given the limitations of employing bulk data and because of contaminating nonmalignant cells and overall leukemia heterogeneity, we investigated differential gene expression in MLLr AMLs using single-cell genomic data. Of the 11 samples sequenced in the AAML1031 cohort, 5 expressed MECOM and 6 did not express MECOM (supplemental Figure 3H). To investigate whether *MECOM* expression within a leukemia correlates with the differentiation phenotype, we examined signatures from nonmalignant HSCs and monocytes within each cell as well as our 122 gene signature that we had defined to be directly regulated by MECOM (Figure 3A-B). HSC signatures were significantly more abundant in AMLs expressing MECOM, whereas monocyte signatures were significantly more abundant in leukemias lacking MECOM. Notably, the gene signature that we defined as being repressed by MECOM was lower in *MECOM*-expressing AMLs (Figure 3C). Furthermore, when performing differential expression analysis between leukemias expressing MECOM or not, we observed that individual HSC-associated genes are upregulated in MECOM-expressing leukemias, whereas monocyte-associated genes and MECOM network genes (none are monocyte-associated genes) are downregulated in leukemias expressing MECOM (Figure 3D). These findings confirm that the phenotypic patterns and gene expression changes that we empirically observed in our cell line models upon acute depletion of MECOM (Figure 2) were recapitulated in primary AMLs.

### Conservation of the MECOM cisRE network in primary AMLs

**Figure 4.**
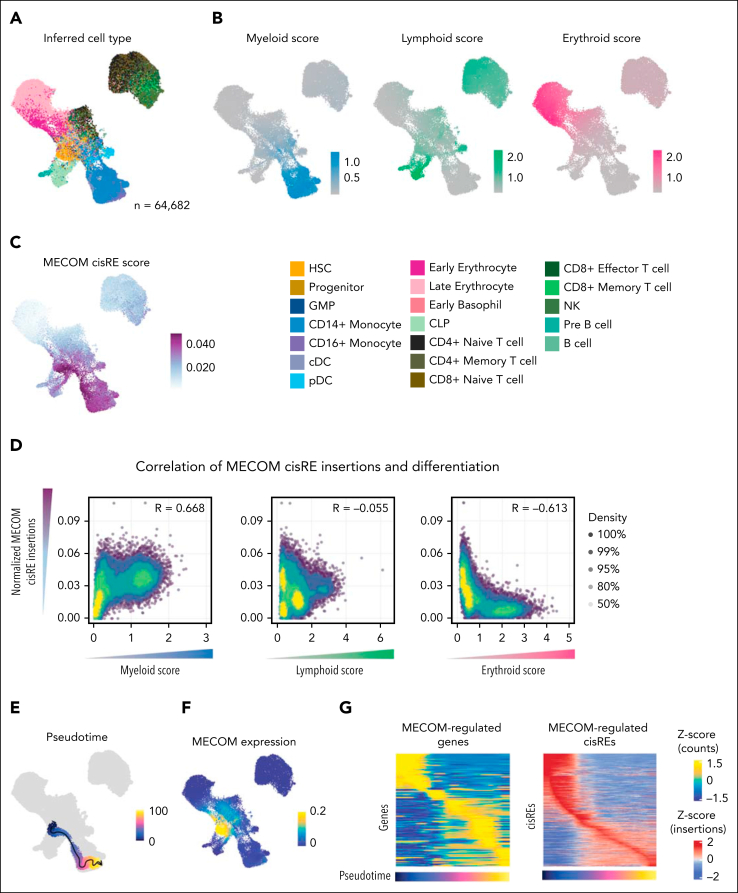
**Direct MECOM chromatin network is repressed in primary leukemia cells.** (A) UMAP of 64 682 cells generated using scATAC-seq from remissions of patients with pediatric AML (n = 20). Cells were colored by predicted cell type derived using label transfer of matching scRNA-seq data. Labels were derived from Lambo et al.^36^ (B) UMAP showing the same cohort as panel A and colored by lineage scores. Lineage scores were calculated as the total insertions in 5000 accessible sites in each lineage, normalized to accessible sites in the other 2 lineages (accessible sites derived from Lambo et al^36^). (C) Cells colored by chromatin accessibility at MECOM-bound loci that were identified to increase in accessibility after the depletion of MECOM. Scores were calculated by ATAC-seq reads at MECOM-bound loci divided by ATAC-seq reads in the TSS and corrected for Tn5 bias. Scores were scaled to the 99th quantile to reduce the effect of outliers. (D) Spearman correlation between lineage scores and MECOM cisRE scores. Each dot represents 1 cell, with cells colored by density. (E) UMAP showing a trajectory inferred using Monocle from inferred HSCs to inferred monocytes. (F) UMAP showing the scaled expression of MECOM in counts from linked scRNA data across cells from remissions. (G) Heat maps showing the scaled expression of MECOM-regulated genes (n = 122) and cisREs (n = 837) along the monocyte trajectory (pseudotime). Each column represents an aggregated minibulk from cells across the inferred pseudotime (100 bins in total). Normalized gene expression scores are derived from linked scRNA samples, with ATAC-seq signal normalized by TSS insertions and Tn5 bias. Both gene expression and ATAC-seq signal were scaled across all cells in the pseudotime. cDC, conventional dendritic cell; CLP, common lymphoid progenitor; GMP, granulocyte-monocyte progenitor; NK, natural killer; pDC, plasmacytoid dendritic cells.

We next investigated whether the chromatin alterations observed in vitro after the acute depletion of MECOM correlate with changes observed due to high MECOM expression in primary AMLs. Given the heterogeneity of chromatin alterations and cell states between different AMLs, we analyzed scATAC-seq of matched remission samples (n = 20 patients) from the AAML1031 cohort, which expressed MECOM and had identifiable trajectories of myeloid, erythroid, and lymphoid differentiation (Figure 4A). To quantify differentiation within the scATAC-seq data, we used peak sets that are specifically accessible in myeloid, lymphoid, and erythroid trajectories and calculated the relative peak accessibility in comparison to other trajectories. This analysis yielded differentiation scores from HSC-like cells to monocytic, mature B cells and late erythroid cells (Figure 4B). For each cell, we then the total number of peaks identified to be repressed by MECOM (MECOM cisRE score), which we identified as being particularly abundant in cells in the HSC-like to monocyte-like axis (Figure 4C). Having calculated MECOM cisRE scores and differentiation scores for each cell, we correlated the scores across all cells and found that accessible chromatin peaks identified to be bound by MECOM were correlated with myeloid differentiation scores, whereas they were anticorrelated with erythroid differentiation scores (Figure 4D). This finding confirms that MECOM-regulated peaks become increasingly accessible during myeloid differentiation. To investigate the peaks in more detail, we inferred a trajectory of differentiation toward monocyte-like populations from cells expressing MECOM using Monocle^41^ (Figure 4E-F). We then correlated the gene expression of putative MECOM target genes in linked scRNA-seq from 20 matched scRNA-seq samples and accessibility of MECOM cis-regulatory regions from scATAC-seq (Figure 4G). We observed substantial decreases in chromatin accessibility and gene expression across the myeloid differentiation trajectory. These analyses in primary leukemia and matched remission samples corroborated MECOM’s role in directly repressing a myeloid differentiation cisRE network.

In addition to these correlative analyses, we sought to empirically validate the conservation of these MECOM-driven gene and cisRE networks in an MLLr cell line model. For this purpose, we examined *MECOM* expression in MLLr AMLs in the Cancer Dependency Map^42^ and found high expression in OCI-AML4, an *MLL*::*ENL* fusion cell model^43^ (supplemental Figure 4A). Therefore, we created an additional CRISPR-engineered, biallelically-tagged MECOM-dTAG isogenic model in OCI-AML4 cells (supplemental Figure 4B) and confirmed that dTAG^V^-1 treatment induced rapid MECOM degradation (supplemental Figure 4C). We then leveraged this model to perform additional bulk RNA-seq and ATAC-seq of dTAG^V^-1–treated OCI-AML4 cells. Differential expression analysis and GSEA confirmed that our repressive MECOM gene and cisRE networks are conserved in this cytogenetically distinct model of MECOM-expressing, high-risk AML (supplemental Figure 4D-I). Overall, our empiric and correlative analyses of MECOM gene regulatory functions illustrate how MECOM acts as a gatekeeper of a highly conserved myeloid differentiation program across diverse high-risk AMLs.

### Functional CRISPRi/a screens identify a *CEBPA* cisRE as a key MECOM-controlled cisRE to block differentiation

**Figure 5.**
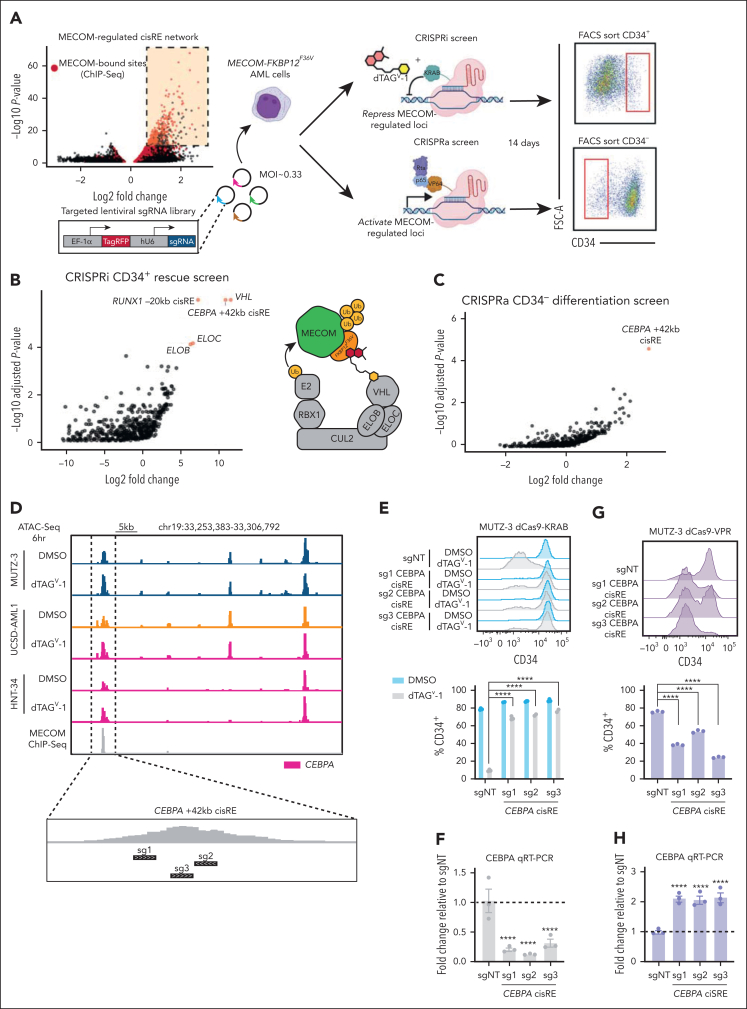
**Functional CRISPR screening identifies CEBPA cisRE as a key regulator of myeloid differentiation in high-risk leukemia.** (A) Schematic overview of the CRISPR screens used to functionally interrogate MECOM-regulated cisREs. An sgRNA oligo library was designed against MECOM-regulated elements (up to 5 sgRNAs per element, depending on the availability of high-quality sgRNA-targeting sites) and packaged into a lentiviral vector. Two different populations of MUTZ-3 MECOM-FKBP12^F36V^ cells were then transduced with this sgRNA library virus at an MOI of ∼0.33, in which one population expressed dCas9-KRAB (CRISPRi screen) and another expressed dCas9-VPR (CRISPRa screen). Cells in the CRISPRi screen were treated with 500 nM dTAG^V^-1 for the duration of the screen. After 14 days of in vitro culture, cells from the CRISPRi and CRISPRa screens were sorted for phenotypically rescued CD34^+^ cells (up-assay) and differentiated CD34^–^ cells (down-assay), respectively. Genomically integrated sgRNAs were sequenced to assess relative sgRNA abundance. Both screens were performed with n = 3 independent replicates. (B-C) Volcano plots depicting sgRNA enrichment/depletion from sorted populations compared to plasmid library DNA (supplemental Table 8). The sgRNA library included sgRNAs targeting the TSSs of *VHL*, *ELOB*, and *ELOC* (5 sgRNAs per gene), which form the E3 ubiquitin-ligase complex recruited by dTAG^V^-1. (D) Genome browser tracks at the *CEBPA* locus encompassing the +42 kb cisRE. ATAC-seq tracks from MECOM-FKBP12^F36V^ cell line models and MECOM ChIP-seq demonstrate increased chromatin accessibility upon dTAG^V^-1 treatment. Three top-scoring *CEBPA* cisRE-targeting sgRNAs were selected for single sgRNA validation experiments. (E) MUTZ-3 dCas9-KRAB cells were infected with sgRNA-expressing lentiviruses targeting either the CEBPA cisRE or a nontargeting sequence. At 48 hours after transduction, cells were treated with 500 nM dTAG^V^-1 vs DMSO. Histogram shows CD34 expression at day 9 (top). Percentage of CD34^+^ cells at day 9 (bottom). n = 3 independent replicates. Mean and SEM are shown. (F) qRT-PCR of *CEBPA* expression in dTAG^V^-1–treated cells 3 days posttreatment. Fold change represents ΔΔCt values compared to the sgNT condition. n = 3 independent replicates. Mean and SEM are shown. Two-sided Student *t* test was used for comparisons. ∗∗∗∗*P* < .0001. (G) MUTZ-3 dCas9-VPR cells were infected with sgRNA-expressing lentiviruses targeting either the CEBPA cisRE or a nontargeting sequence. Histogram shows CD34 expression at day 9 (top). Percentage of CD34^+^ cells at day 9 (bottom). n = 3 independent replicates. Mean and SEM are shown. (H) qRT-PCR of *CEBPA* expression in all conditions 3 days posttransduction. Fold change represents ΔΔCt values compared to the sgNT condition. n = 3 independent replicates. Mean and SEM are shown. Two-sided Student *t* test was used for comparisons. ∗∗∗∗*P* < .0001. FACS, fluorescence-activated cell sorted; FSC-A, forward scatter area; MOI, multiplicity of infection; qRT-PCR, quantitative reverse transcription polymerase chain reaction; sgNT, nontargeting sgRNA.

Having defined and validated a conserved myeloid differentiation gene network repressed by MECOM, we next sought to pinpoint critical nodes in this network. Functional genomic screens were employed to identify MECOM-controlled cisREs that are essential in facilitating MECOM’s ability to block differentiation in stem cell–like leukemia cells. We designed a lentiviral single-guide RNA (sgRNA) library of 2741 sgRNAs targeting MECOM-repressed cisREs and employed it in both a CRISPR inhibition^44^ (CRISPRi) rescue screen and a CRISPR activation^45^ (CRISPRa) differentiation screen in MUTZ-3 AML progenitor cells (Figure 5A). First, in the CRISPRi screen, we treated deactivated-Cas9 (dCas9)-KRAB–expressing cells with dTAG^V^-1 and simultaneously repressed individual MECOM-regulated cisREs with KRAB to investigate whether the repression of any single cisRE was sufficient to maintain leukemia cells in a CD34^+^ stem cell–like state in the absence of endogenous MECOM. After 2 weeks of culture, we sequenced integrated sgRNAs in the CD34^+^ phenotypically rescued population (supplemental Figure 5A) and identified a strong enrichment of positive control sgRNAs targeting the transcription start sites (TSSs) of *VHL*, *ELOB*, and *ELOC* (Figure 5B). As anticipated, the knockdown of VHL or the ELOB-ELOC subcomplex^46^ renders dTAG^V^-1, a VHL-targeting PROTAC, inactive. Thus, cells expressing these sgRNAs are resistant to dTAG^V^-1-mediated degradation and retain a stem cell–like phenotype, characterized by sustained CD34 expression. We also identified a significant enrichment of sgRNAs targeting both a cisRE 20 kilobase (kb) upstream from the *RUNX1* TSS (*RUNX1* –20 kb) and a cisRE 42 kb downstream from *CEBPA* TSS (*CEBPA* +42 kb) (Figure 5B; supplemental Table 8). In an orthogonal interrogation of cisRE function, we next investigated whether the activation of any single MECOM-repressed element, in the absence of MECOM perturbation, is sufficient to induce differentiation of stem cell–like leukemia cells. To accomplish this, we engineered MUTZ-3 cells to constitutively express the targetable transcriptional activator dCas9-VPR and transduced them with the same MECOM cisRE-targeting sgRNA library. Rather than sorting for CD34^+^ cells, in this screen, we flow cytometrically sorted and sequenced sgRNAs from CD34^–^ cells undergoing myeloid differentiation (Figure 5A). Notably, the only significant hit from this CRISPRa prodifferentiation screen was the same *CEBPA* +42 kb cisRE (Figure 5C; supplemental Table 8). Given that this *CEBPA*-linked cisRE was the strongest hit from both screens and has been previously implicated in myeloid differentiation,^47^ we selected this target for further validation. For single sgRNA studies, we used our top 3 performing *CEBPA* cisRE-targeting sgRNAs (Figure 5D) and compared them to a nontargeting control sgRNA. Consistent with our pooled screening results, KRAB-mediated repression of the *CEBPA* +42 kb cisRE resulted in decreased *CEBPA* expression and maintained leukemia cells in a CD34^+^ stem cell–like state following MECOM degradation (Figure 5E-F; supplemental Figure 5B). Furthermore, the activation of this cisRE alone was sufficient to increase *CEBPA* expression and, notably, drive the differentiation of AML cells without disrupting endogenous MECOM function (Figure 5G-H; supplemental Figure 5C).

We next characterized the dynamics of chromatin accessibility at this *CEBPA* cisRE during myeloid differentiation in primary leukemia cells (supplemental Figure 5D). We divided the remission samples from the AAML1031 cohort into clusters (supplemental Figure 5E) to compare chromatin accessibility within identified cisREs around *CEBPA* and approximated both *CEBPA* and *MECOM* activity along the pseudotime trajectory using gene expression and transcription factor motif enrichments from the single-cell genomic data (supplemental Figure 5F-H). As expected, we observed a gradual increase in both the expression and motif enrichment of CEBPA and a gradual decrease in both the expression and motif enrichment of MECOM over the course of myeloid differentiation. We then investigated the accessibility of the *CEBPA* +42 kb cisRE across all defined differentiation clusters (supplemental Figure 5I) and found that accessibility was generally restricted to early and later myeloid lineages (supplemental Figure 5J). All these data suggest that the cisREs regulated by MECOM are both specific and required for myeloid lineage specification. Overall, our functional screens and validation suggest a previously unappreciated and surprisingly simple regulatory logic underlying MECOM’s role in promoting stem cell–like states in AML through the repression of a single critical cisRE.

### Repression of the *CEBPA* +42 kb cisRE prevents myeloid differentiation induced by MECOM perturbation in primary leukemia cells

After validating the function of the CEBPA +42 kb cisRE in stem-like AML cells, we tested its necessity in primary AMLs. Given the inability to create stable degron models in these sensitive, heterogeneous primary samples,^48^^,^^49^ we engineered a novel CRISPR-Cas9 nuclease (Cas9n)-based strategy to disrupt *CEBPA* cisRE function. We hypothesized that a dual-sgRNA approach with Cas9n could be leveraged to induce a DNA microdeletion proximal to the summit of the *CEBPA* cisRE ATAC-seq peak and corresponding MECOM ChIP-seq peak (supplemental Figure 6A) to permanently inactivate the element. These sgRNAs could be codelivered with an sgRNA targeting the coding sequence of *MECOM* to induce simultaneous inactivation of the *MECOM* locus and *CEBPA* cisRE. We initially tested this approach in MUTZ-3 MECOM-dTAG cells by electroporating them with Cas9 protein and chemically synthesized sgRNAs targeting the +42 kb *CEBPA* cisRE and *MECOM* coding sequence (CDS) or *AAVS1* safe harbor locus. This strategy efficiently conferred a 37-bp microdeletion in the *CEBPA* cisRE as well as created small insertions/deletions at the *MECOM* and *AAVS1* loci (supplemental Figure 6B). Targeting the *MECOM* coding sequence caused near complete loss of stem cell–like CD34^+^ MUTZ-3 cells, which was completely rescued by *CEBPA* cisRE inactivation (supplemental Figure 6C-D). The validation of this “degron-free” Cas9n-mediated engineering strategy instilled confidence that this approach could be employed to study the relationship between MECOM and the *CEBPA* cisRE in the setting of short-term stromal cell cocultures of these primary AML cells.

**Figure 6.**
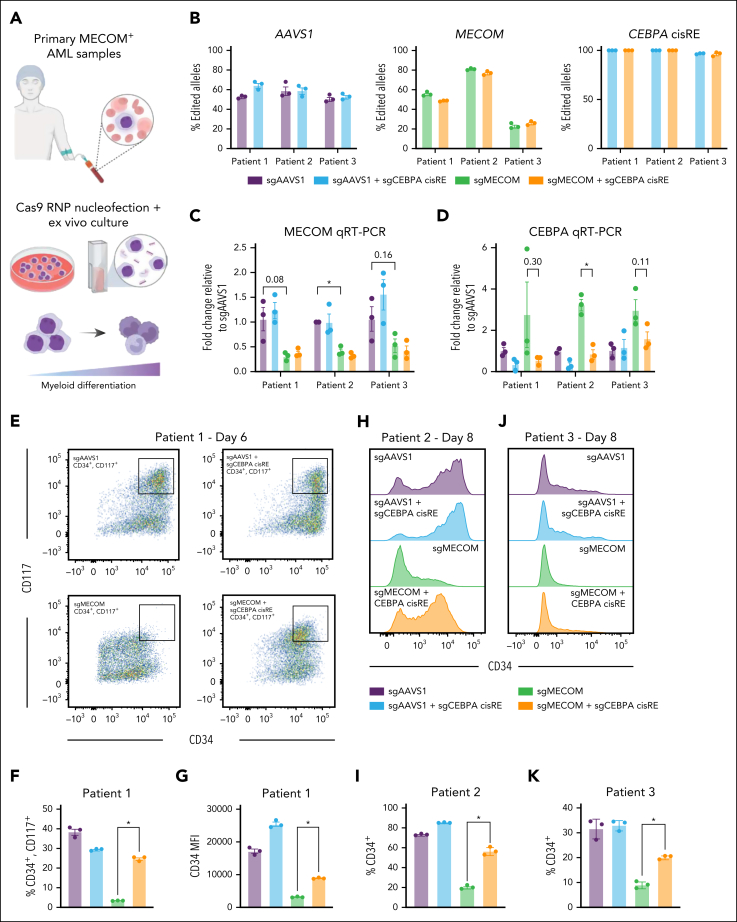
**CEBPA cisRE is necessary for differentiation of MECOM-driven AML cells.** (A) Primary MECOM^+^ AML cells were harvested from patients at diagnosis and cryopreserved (supplemental Table 9). Cells were thawed for short-term ex vivo culture and electroporated with CRISPR-Cas9 RNPs to induce genetic perturbations at the *MECOM* vs *AAVS1* locus ± *CEBPA* +42 kb cisRE. (B) Efficiency of gene editing in 3 biologically distinct primary AMLs at the *AAVS1*, *MECOM*, and *CEBPA* (cisRE) loci. Editing estimated using Sanger sequencing of amplicons followed by sequence trace decomposition analysis with Inference of CRISPR Edits (ICE) tool.^58^ For *CEBPA* cisRE, only deletions resulting from dual guide cleavage were counted. n = 3 technical replicates. Mean and SEM are shown. (C-D) qRT-PCR of *CEBPA* and *MECOM* expression in all conditions 3 days postelectroporation. Fold change represents ΔΔCt values compared to the sgNT condition. n = 3 technical replicates, and mean and SEM are shown. n = 3 independent replicates, and mean and SEM are shown. Two-sided Student *t* test was used for comparison. ∗*P* < .05. (E-G) Immunophenotypic analysis of a primary leukemia sample (patient 1, supplemental Table 9) 6 days postelectroporation. (E) Bivariate plot showing CD34 and CD117 expression assessed by flow cytometry. Black box denotes CD34^+^/CD117^+^ subset. (F) Percentage of CD34^+^/CD117^+^ cells. (G) CD34 expression measured by MFI. n = 3 independent technical replicates. Mean and SEM are shown. A Mann-Whitney test was used for comparisons. ∗*P* < .05. (H-I) Immunophenotypic analysis of a primary leukemia sample (patient 2, supplemental Table 9) 8 days postelectroporation. (H) Histogram showing CD34 expression assessed by flow cytometry. (I) Percentage of CD34^+^ cells. n = 3 independent technical replicates. Mean and SEM are shown. A Mann-Whitney test was used for comparisons. ∗*P* < .05. (J-K) Immunophenotypic analysis of primary leukemia (patient 3, supplemental Table 9) 8 days postelectroporation. (J) Histogram showing CD34 expression assessed by flow cytometry. (K) Percentage of CD34^+^ cells. n = 3 independent replicates. Mean and SEM are shown. Two-sided Student *t* test was used for comparisons. ∗*P* < .05. MFI, mean fluorescence intensity; sgNT, nontargeting sgRNA.

We successfully edited *MECOM* and the *CEBPA* cisRE in 3 primary AMLs expressing high levels of *MECOM* (Figure 6A-B; supplemental Table 9). A reduction in *MECOM* expression was confirmed in the *MECOM*-edited samples, consistent with nonsense-mediated messenger RNA decay^50^ (Figure 6C). Moreover, across all samples, *MECOM*-editing induced a significant increase in *CEBPA* expression, which was almost completely prevented by the inactivation of the *CEBPA* cisRE (Figure 6D). Remarkably, *MECOM* perturbations induced significant loss of stem cell–like leukemia cells, as demonstrated by the loss of surface markers CD34 and/or CD117 across all patient samples (Figure 6E-K; supplemental Figure E-F), whereas the inactivation of the *CEBPA* cisRE could significantly rescue this differentiation phenotype and maintain cells in more stem cell–like states. Furthermore, for 1 sample that grew in culture, *CEBPA* cisRE inactivation prevented the observed transient increase in cell growth following *MECOM* perturbation (supplemental Figure 6G). Overall, our cisRE inactivation strategy has demonstrated the functional conservation of a key MECOM-regulated cisRE linked to *CEBPA* and demonstrated its necessity for the differentiation of primary AMLs.

### Transient activation of the *CEBPA* +42 kb cisRE promotes differentiation of primary AML cells and reduces leukemic burden in vivo

**Figure 7.**
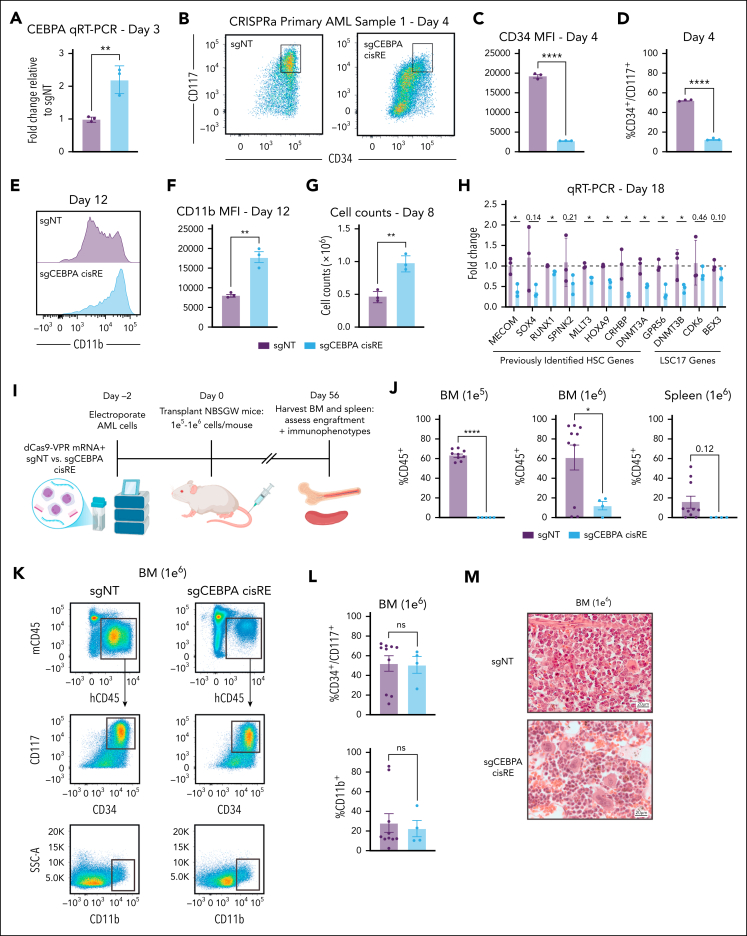
**Transient activation of CEBPA cisRE is sufficient to differentiate high-risk, stem cell–like AML cells.** (A) qRT-PCR of *CEBPA* expression 3 days postelectroporation. Fold change represents ΔΔCt values compared to the sgNT condition. n = 3 independent replicates. Mean and SEM are shown. Two-sided Student *t* test was used for comparison. ∗∗*P* < .01. (B-D) Immunophenotypic analysis of a primary leukemia sample (patient 1, supplemental Table 9) 4 days postelectroporation. (B) Bivariate plot showing CD34 and CD117 expression assessed by flow cytometry. Black box denotes CD34^+^/CD117^+^ subset. (C) CD34 expression measured by MFI. (D) Percentage of CD34^+^/CD117^+^ cells. n = 3 independent replicates. Mean and SEM are shown. Two-sided Student *t* test was used for comparisons. ∗∗∗∗*P* < .0001. (E-F) Immunophenotypic analysis of a primary leukemia sample (patient 1, supplemental Table 9) 12 days postelectroporation. (E) Histogram showing CD11b expression assessed by flow cytometry. (F) Percentage of CD11b^+^ cells. n = 3 independent replicates. Mean and SEM are shown. Two-sided Student *t* test was used for comparison. ∗∗ *P* < .01. (G) Viable cell counts by trypan blue exclusion in primary leukemia sample (patient 1, supplemental Table 9) 8 days postelectroporation. n = 3 independent replicates. Mean and SEM are shown. Two-sided Student *t* test was used for comparison. ∗∗ *P* < .01. (H) qRT-PCR data of a panel of established HSC genes and LSC17 genes 18 days postelectroporation demonstrating the robust differentiation of a primary leukemia sample (patient 1, supplemental Table 9) following transient activation of *CEBPA* cisRE. n = 3 independent replicates. Mean and SEM are shown. Two-sided Student *t* test was used for comparison. ∗ *P* < .05. (I) Schematic of the experiment to assess the in vivo impact of *CEBPA* cisRE activation of a xenotransplanted primary leukemia sample (patient 1, supplemental Table 9). Cells were electroporated with mRNA encoding dCas9-VPR and 2 chemically synthesized sgRNAs targeting the *CEBPA* cisRE or a sgNT. Cells recovered in ex vivo culture for 2 days postelectroporation and then injected via the tail vein. All animals were euthanized 56 days after transplant for analysis of leukemia burden in spleens and BM. (J) Quantification of human cell chimerism (hCD45^+^) in the BM of mice transplanted with 1 × 10^5^ to 1 × 10^6^ cells and spleens of mice transplanted with 1 × 10^6^ cells. n = 4 to 10 xenotransplant recipients as shown. Mean and SEM are shown. Two-sided Student *t* test was used for comparison. ∗∗∗∗ *P* < .0001, ∗ *P* < .05. (K-L) Immunophenotypic analysis of the BM of mice transplanted with 1 × 10^6^ cells. Cells were labeled with a cocktail of antibodies including mouse CD45 and human CD45, CD34, CD117, and CD11b. (K) Bivariate plots depicting the gating strategy for quantification of engrafted leukemia stem/progenitor cells (CD34^+^/CD117^+^) and mature cells (CD11b^+^). Black boxes denote human cell subset (top), CD34^+^/CD117^+^ subset (middle), and CD11b^+^ subset (bottom). (L) Percentage of CD34^+^/CD117^+^ cells (top) and CD11b^+^ cells (bottom). n = 4 to 10 xenotransplant recipients as shown. Mean and SEM are shown. Two-sided Student *t* test was used for comparison. (M) Hematoxylin and eosin (H&E) staining of bone marrow of mice transplanted with 1 × 10^6^ cells. BM, bone marrow; MFI, mean fluorescence intensity, mRNA, messenger RNA; ns, not significant; sgNT, nontargeting sgRNA.

Given the conserved role of the *CEBPA* cisRE in blocking differentiation of high-risk AMLs, we assessed whether the activation of this regulatory node alone is sufficient to induce differentiation. To test this, we codelivered in vitro-transcribed CRISPRa (dCas9-VPR) messenger RNA and 2 chemically synthesized sgRNAs targeting the *CEBPA* cisRE or a nontargeting sgRNA into primary AML cells. Treated cells were maintained in ex vivo culture and monitored for signs of immunophenotypic differentiation. Quantitative reverse transcription polymerase chain reaction analysis revealed that *CEBPA* cisRE targeting by CRISPRa conferred a twofold increase in *CEBPA* expression (Figure 7A). Furthermore, we observed striking differentiation phenotypes with the loss of stem cell surface markers CD34 and CD117 (Figure 7B-D) and an increase in expression of the mature myeloid cell marker CD11b (Figure 7E-F). Notably, these differentiation phenotypes were observed across 3 different patient samples (supplemental Figure 7A-D; supplemental Table 9). In a sample that successfully grew in culture, we also observed a marked increase in growth after CRISPRa treatment, another independent indicator of a differentiation or cell proliferation phenotype being induced^51^^,^^52^ (Figure 7G). To orthogonally evaluate the robustness of cell-state changes induced by *CEBPA* cisRE activation, we performed quantitative reverse transcription polymerase chain reaction analysis after 18 days of culture post-editing to assess the expression of a panel of bona fide stem cell genes. This analysis revealed that *CEBPA* cisRE activation reduced the expression of established HSC genes^40^^,^^53^ as well as clinically relevant LSC17 genes^54^ (Figure 7H). Notably, *CEBPA* cisRE activation resulted in reduced expression of MECOM itself, showing how the reactivation of myeloid differentiation is sufficient to overcome the promotion of stem cell gene expression programs.

Finally, we performed xenotransplantation of electroporated primary AML cells into nonirradiated immunodeficient NBSGW mice to assess how *CEBPA* cisRE activation impacts leukemia burden and engraftment of modified cells (Figure 7I). Notably, this patient-derived xenotransplant model was established using primary 3q26 AML cells, a subtype for which there are extremely few reported cases of successful model generation.^19^ Across 2 cell doses, at 8 weeks posttransplant, *CEBPA* cisRE-activated AML cells either did not engraft in any mice (1 × 10^5^ cell dose: 0/5 mice) compared with 100% engraftment of controls (9/9 mice) or engrafted with significantly lower human chimerism in the bone marrow and spleens (1 × 10^6^ cell dose) compared with nontargeting controls (12.1% vs 85.5%, bone marrow hCD45^+^, *P* < .05) (Figure 7J; supplemental Figure 7E). Furthermore, the estimated leukemia-initiating cell frequency, calculated based on the frequencies of human cell engraftment in the bone marrow^55^ of *CEBPA* cisRE-activated AML cells, was significantly lower than nontargeting controls (1/910 241 (0.00 011%) vs 1/251 838 (0.00 040%), *P* = .037) (supplemental Figure 7F). We also observed an average 1.73-fold increase in spleen weights of mice transplanted with control cells compared with mice transplanted with *CEBPA* cisRE-activated cells (supplemental Figure 7G-H). Notably, the lower number of cells in the *CEBPA* cisRE-activated group that did engraft in mice conferred a mostly stem cell–like immunophenotype (CD34^+^, CD117^+^, CD11b^–^) similar to those observed in controls, suggesting that these cells might escape CRISPRa activity and retain their phenotype (Figure 7K-L). The analysis of fixed hematoxylin and eosin–stained bone marrow sections confirmed substantial human AML xenografts in mice transplanted with control cells (Figure 7M). In contrast, mice transplanted with *CEBPA* cisRE-activated cells showed a marked reduction in leukemia burden and a high frequency of multinucleated giant cells (Figure 7M). In summary, these results underscore the utility of reactivating myeloid differentiation programs in high-risk leukemia to significantly disrupt the fitness of stem cell–like leukemia cells in vivo.

## Discussion

A direct understanding of how stem cell gene regulatory programs are co-opted in aggressive AMLs is critical for developing targeted therapies. Here, we investigate how elevated MECOM expression, a hallmark of high-risk AMLs, enables these programs. Motivated by clinical findings, including MECOM insertions driving leukemias in gene therapy trials,^56^ we used targeted MECOM degradation with functional genomic readouts to reveal that MECOM primarily acts as a transcriptional repressor, suppressing differentiation programs (a previously underappreciated function).^13^, ^14^, ^15^, ^16^, ^17^

Through functional genomic perturbations and high-throughput screens, we identified key regulatory nodes within MECOM-regulated gene networks. Surprisingly, despite MECOM’s widespread chromatin regulation, repression of a single *CEBPA* cisRE is both necessary and sufficient to sustain its role in blocking myeloid differentiation. These findings highlight the power of functional screens in uncovering therapeutic vulnerabilities.

Given that the reactivation of a single *CEBPA* regulatory element can induce myeloid differentiation and reduce leukemic burden, our findings raise the intriguing possibility that differentiation-based therapies could also be leveraged in MECOM-high AMLs. Although our approach focused on genetic and epigenetic manipulation of the CEBPA cisRE, pharmacologic strategies that promote myeloid maturation, such as all-trans retinoic acid or histone deacetylase inhibitors, may offer complementary or alternative avenues for differentiation induction in this context. Although all-trans retinoic acid has shown limited success in non-APL AMLs, its efficacy may improve in molecularly defined subgroups, such as those with MECOM overexpression.^57^ Future studies aimed at testing these compounds in preclinical models of MECOM-driven AML may provide translational insight and help prioritize differentiation therapies for this high-risk AML subtype.

This work establishes proof-of-concept for differentiation-based therapy in high-risk AMLs by reactivating *CEBPA*, rather than solely eradicating stem-like populations. As observed in APL, this approach could provide a viable strategy for otherwise incurable AMLs.

Conflict-of-interest disclosure: V.G.S. serves as an adviser to Ensoma, Cellarity, and Beam Therapeutics, unrelated to the present work. K.S. received grant funding from the Dana-Farber Cancer Institute/Novartis Drug Discovery Program and is a member of the scientific advisory board and has stock options with Auron Therapeutics on topics unrelated to the present work. The remaining authors declare no competing financial interests.

The current affiliation for R.A.V. is UT Southwestern Medical Center, Dallas, Texas.

## Supplementary Material

Supplemental Methods, Figures, Table Legends, and References
