# Supplementary material for: CEBPA repression by MECOM blocks differentiation to drive aggressive leukemias
Source: Blood. 2025 Sep 26;146(25):3019–35. doi: 10.1182/blood.2025028954 (PMC12824682; doi:10.1182/blood.2025028954)
Supplement: Supplemental Methods, Figures, Table Legends, and References [file BLOOD_BLD-2025-028954-mmc1.pdf]

SUPPLEMENTARY MATERIAL

METHODS

EXPERIMENTAL MODEL AND STUDY PARTICIPANT DETAILS

Lead contact

Further information and reagent requests should be directed to and will be fulfilled by the Lead Contact Vijay G. Sankaran (sankaran@broadinstitute.org).

Materials availability

All unique materials will be available upon request to the lead contact.

Data reporting

No statistical methods were used to predetermine sample sizes but our sample sizes are similar to those reported in previous publications[1,2]. Data collection and analysis were not performed blind to the conditions of the experiments. No animals or data points were excluded from analysis.

Data and code availability

All processed and raw sequencing data are deposited in National Center for Biotechnology Information Gene Expression Omnibus at the following GEO accession numbers: GSE284553 (reviewer access token: ejifegkknfaxrux) for MUTZ-3, UCSD-AML1, HNT-34, and OCI-AML4 bulk ATAC-seq data; GSE284555 (reviewer access token: ilszkussjdwtlyb) for MECOM(HA) and H3K27ac ChIP-seq data in MUTZ-3 cells; GSE284556 (reviewer access token: atavkwamplebrol) for MUTZ-3 PRO-seq data; and GSE284558 (reviewer access token: chklomygxhyxtir) for MUTZ-3, UCSD-AML1, HNT-34, and OCI-AML4 bulk RNA-seq data. Primary AML bulk RNA-seq, scRNA-seq, and scATAC-seq datasets from Lambo et al 2023[3] can be accessed at the following GEO accession numbers: GSE235063, GSE235308.

Cell line and primary AML cell culture

MUTZ-3 cells (DSMZ), HNT-34 cells (Creative Bioarray), and OCI-AML4 cells (DSMZ) were cultured at 37 °C in α-MEM (Life Technologies) supplemented with 20% FBS, 20% conditioned medium from 5637 cells[4] (ATCC) and 1% penicillin/streptomycin. Confluency for all cells was maintained between 7×10^5 and 1.5×10^6 ml^{-1}.

UCSD-AML1 cells (a gift from Dr. Kimberly Stegmaier's lab) were cultured at 37 °C in RPMI 1640 (Life Technologies) supplemented with 20% FBS, 1% penicillin/streptomycin and 10ng/mL GM-CSF (Peprotech). Confluency was maintained between 5×10^5 and 1.5×10^6 ml^{-1}.

293T cells were cultured at 37 °C in DMEM (Life Technologies) supplemented with 10% FBS and 1% penicillin/streptomycin.

MS-5 cells, prior to co-culture, were cultured at 37 °C in α-MEM (Life Technologies) supplemented with 10% FBS, 2 mM L-glutamine and 2 mM sodium pyruvate. Confluency was maintained at <95% and split in culture 1:3 every 3 days. Cells were maintained at low passage number (<12).

Primary AML cells were collected with informed consent according to procedures approved by either the University Health Network (UHN) or Boston Children's Hospital and Dana Farber Cancer Institute's Research Ethics Boards. Two days prior to thawing primary AML cells, MS-5 cells were plated at ~50% confluency in 12-well or 6-well plates. Primary AML cells were then thawed and immediately placed in co-culture with MS-5 cells. Cells were co-cultured at 37 °C in IMDM (Life Technologies) supplemented with 10% FBS, 2% L-glutamine, CC100 cytokine cocktail (Stem Cell Technologies), and 100 ng/ml TPO at concentrations between 1 and 2.5×10^6 ml^{-1}.

Mouse model

NOD.Cg-$Kit^{W\text{-}41J}$ Tyr^{+} $Prkdc^{scid}$ $Il2rg^{tm1Wjl}$ (NBSGW) mice were obtained from the Jackson Laboratory (stock 026622)[5]. Littermates of the same sex were randomly assigned to experimental groups. NBSGW were interbred to maintain a colony of animals homozygous or hemizygous for all mutations of interest. The Institutional Animal Care and Use Committee at Boston Children's Hospital approved the study protocol and provided guidance and ethical oversight.

Lentiviral production

For lentiviral production, 293T cells were expanded to reach 80% confluency per plate on the day of transfection. 1–20 10 cm^2 plates were prepared per lentiviral construct. For each plate, 4 μg of psPAX2 packaging plasmid, 2 μg of pMD2.G envelope plasmid, and 8 μg of sgRNA vector construct was mixed in Opti-MEM media (Gibco, 31985-062). This mix was then diluted in Lipofectamine 3000 and combined with P3000 reagent per the manufacturer's protocol and added dropwise to cells. 12-16 hours later, 293T medium was removed and changed to DMEM with 20% FBS and 1% penicillin/streptomycin. 24 hours later the media was harvested and filtered through a Stericup 0.45 mm PVDF membrane (Millipore, SCHVU01RE), and transferred to ultra-clear centrifuge tubes (Beckman Coulter, 344058). Virus was subsequently concentrated using a Beckman Coulter SW32Ti Ultracentrifuge with the following parameters: Speed: 24,000 rpm, time: 1 hour and 30 minutes, Temperature: 4C, maximum acceleration and deceleration 9. The supernatant was removed, and the virus pellet was resuspended with the appropriate media. Concentrated virus was stored at -80ºC until further usage.

Lentiviral transduction

Cells were transduced at a density of 500,000-1 million cells per mL. Concentrated virus was added to cells along with 8 µg/mL polybrene (Sigma Aldrich, TR-1003-G). Cells were then spinfected at 2,000 rpm for 90 mins at 37ºC. 12-16 hours after spinfection, the media was replaced by the appropriate complete media.

Transplantation assays

Primary AML cells were thawed and plated onto an MS-5 co-culture with primary cell medium (see methods) to recover for 24 hours. Cells were then electroporated and placed back into co-culture. 48 hours later, non-irradiated NBSGW mice (between 8-10 weeks of age) were tail vein injected with modified primary AML cells (1×10^5 - 1×10^6 cells). Peripheral human chimerism was assessed at 4 and 7 weeks and animals were sacrificed at 8 weeks for bone marrow (BM) and spleen. Human chimerism and corresponding immunophenotypes were assessed by flow cytometry. The relative percentages of human chimerism and cell counts were used in conjunction to quantify the size (cellular quantity) of human leukemia cell xenografts in the BMs and spleens. Functional leukemia initiating cell (LIC) frequencies were calculated based on human cell engraftment frequencies in the bone marrow of transplanted mice using the ELDA software[6]. Engraftment of leukemia cells was considered to have occurred if human cell chimerism in the bone marrow was >5%. A piece of spleen and a whole femur from each mouse was fixed in Bouin's fixative solution for over 24 hours, followed by two consecutive washes in 70% ethanol. Subsequently, bones were decalcified with formic acid, samples were embedded in paraffin, 4 µm sectioned and H&E stained at the Rodent Histopathology Core at Harvard Medical School. Stained slides were analyzed on Zeiss Axio Imager Z2 Microscope at the Cellular Imaging Core Facility Boston Children's Hospital.

Flow cytometry and cell sorting

MUTZ-3 and UCSD-AML1 cells were stained with anti-CD34-APC (BioLegend, 343607). MUTZ-3 cells were also stained with anti-CD14-PE-Cy7 (BioLegend, 367112). *Ex-vivo* cultured Primary AML samples for were stained with anti-CD34-Alexa Fluor® 488 (BioLegend, 343518), anti-CD117-PE (BioLegend, 313204), and/or anti-CD11b-PE-Cy7 (BioLegend, 101216). Xenotransplant samples were stained with anti-human CD34-BV421 (BioLegend, 343610), anti-human CD117-PE (BioLegend, 313204), anti-human CD11b-PE-Cy7 (BioLegend, 101216), anti-human CD45-APC (BioLegend, 304037), and anti-mouse CD45-FITC (BioLegend, 103108). Two microliters of each antibody were used per 1×10^5 cells in 100 µl in all experiments.

Flow cytometric analyses were conducted on a BD LSRII, LSR Fortessa or Accuri C6 instruments and all data were analyzed using FlowJo software (v.10.10). FACS was performed on a BD Aria in a sterile biosafety cabinet where samples were collected in PBS containing 2% FBS and subsequently replated in the appropriate human cell culture medium. Alternatively, for molecular analyses of sorted populations (RT-qPCR or gDNA PCR for CRISPR screens) cells were sorted

into Eppendorf or conical tubes containing Buffer RLT Plus (QIAGEN) with 1% BME and immediately frozen at −80 °C for downstream analyses.

Cell cycle analysis

MUTZ-3-dTAG and UCSD-AML1-dTAG cells were treated with 500nM dTAG^{V}-1 or DMSO for 48 hours, incubated with 5-ethynyl-2′-deoxyuridine (EdU) (Thermo Fisher Scientific, C10636), then stained with a fluorescent CD34 antibody. Cells were then fixed, permeabilized per the manufacturer's recommendations and incubated with propidium iodide (PI) to stain total DNA content. Flow cytometry analysis ran at low speeds (<400 events/second) was then performed to assess cell cycle states.

Western blot analysis

Total protein lysate of cells was extracted by RIPA buffer in presence of protease inhibitor cocktail on ice for 30 minutes. The total lysate was then linearized by 1X SDS loading buffer and heated at 55 °C for 10 minutes. The lysate was loaded onto 4-20% Mini-PROTEAN TGX Precast Protein Gels (BioRad, 456104) before being transferred to a PVDF membrane using BioRad Trans-Blot Turbo Transfer System. The membrane was blocked in LI-COR Intercept Blocking Buffer and incubated with primary antibodies at 1:1000 dilution in LI-COR Intercept Antibody Diluent at 4 °C overnight, then washed and incubated in 1:1000 anti-mouse-HRP secondary antibody for 90 minutes at room temperature. Membrane was developed using BioRad Clarity Western ECL Substrate and Reagent (BioRad, 1705061) and imaged with BioRad system.

Bulk RNA-seq

Total RNA was extracted using the RNeasy Micro kit (QIAGEN, 74004) from 10,000-50,000 cells sorted or harvested in 25 µl Buffer RLT Plus with 1% BME. Then we proceeded with the SmartSeq2 protocol from the reverse transcription step using 10 ng of RNA[7]. The whole transcriptome amplification step was set at ten cycles. Bulk RNA libraries were pooled at appropriate molar ratios to obtain at least 20 million reads per library. Libraries were subject to paired-end sequencing using NextSeq550 High Output kits with 150 cycles.

ATAC-seq

Accessible chromatin was assessed using Assay for Transposase-Accessible Chromatin using sequencing (ATAC-seq) as previously described with the Omni-ATAC protocol[8] with minor adaptations detailed here. *MECOM-FKBP12^{F36V}* modified MUTZ-3, UCSD-AML1, HNT-34 and OCI-AML4 cells were treated with 500nM dTAG^{V}-1 or DMSO for 6 hours and then 50,000 live cells were sorted into PBS with 2% BSA. Cells were washed twice in 150µL and 50µL 1xPBS, resuspended in 50µL ATAC-seq lysis buffer, incubated for 10 min on ice and centrifuged at 400g for 10 min at 4 °C. The pellet was incubated in the transposase reaction mix (25µL 2×TD buffer

(Illumina), 2.5µL transposase (Illumina Cat# FC-121-1030) and 22.5µL nuclease-free water) for 30min at 37 °C with gentle agitation. After DNA purification with the Zymo DNA Clean and Concentrator Kit (Zymo, Cat# D4033) libraries were amplified with NEBNext High-Fidelity PCR Master Mix (NEB, Cat# M0541S) using custom Nextera primers. Libraries for sequencing were size selected with Agencourt AMPure XP beads (Beckman Coulter, Cat# A63880). DNA concentration was measured with an Invitrogen Qubit fluorometer (Life Technologies) and Agilent Fragment Analyzer. The libraries were sequenced using the Illumina NextSeq 500 platform and the 75-bp paired-end configuration to obtain at least 30 million reads per sample.

PRO-seq

Aliquots of frozen (-80C) permeabilized MUTZ-3 cells were thawed on ice and pipetted gently to fully resuspend. Aliquots were removed and permeabilized cells were counted using a Luna II, Logos Biosystems instrument. For each sample, 1 million permeabilized cells were used for nuclear run-on, with 50,000 permeabilized Drosophila S2 cells added to each sample for normalization. Nuclear run on assays and library preparation were performed essentially as described in Reimer et al. 2021[9] with modifications noted: 2X nuclear run-on buffer consisted of (10 mM Tris (pH 8), 10 mM MgCl2, 1 mM DTT, 300mM KCl, 20uM/ea biotin-11-NTPs (Perkin Elmer), 0.8U/uL SuperaseIN (Thermo), 1% sarkosyl). Run-on reactions were performed at 37C. Adenylated 3' adapter was prepared using the 5' DNA adenylation kit (NEB) and ligated using T4 RNA ligase 2, truncated KQ (NEB, per manufacturer's instructions with 15% PEG-8000 final) and incubated at 16C overnight. 180uL of betaine blocking buffer (1.42g of betaine brought to 10mL with binding buffer supplemented to 0.6 uM blocking oligo (TCCGACGATCCCACGTTCCCGTGG/3InvdT/)) was mixed with ligations and incubated 5 min at 65C and 2 min on ice prior to addition of streptavidin beads. After T4 polynucleotide kinase (NEB) treatment, beads were washed once each with high salt, low salt, and blocking oligo wash (0.25X T4 RNA ligase buffer (NEB), 0.3uM blocking oligo) solutions and resuspended in 5' adapter mix (10 pmol 5' adapter, 30 pmol blocking oligo, water). 5' adapter ligation was per Reimer et al. 2021 but with 15% PEG-8000 final. Eluted cDNA was amplified 5-cycles (NEBNext Ultra II Q5 master mix (NEB) with Illumina TruSeq PCR primers RP-1 and RPI-X) following the manufacturer's suggested cycling protocol for library construction. A portion of preCR was serially diluted and for test amplification to determine optimal amplification of final libraries. Pooled libraries were sequenced using the Illumina NovaSeq platform.

ChIP-seq

Chromatin immunoprecipitation followed by sequencing (ChIP-seq) was performed on chromatin from 1×10^6 $CD34^+$ MUTZ-3-dTAG cells after treatment with 500nM $dTAG^V$-1 or DMSO for 6 hours. Cells were cross-linked with 1% methanol-free formaldehyde (Pierce Life Technologies, 28906), quenched with 0.125 M glycine and frozen at −80 °C and stored until further processing. ChIP reaction was performed with iDeal ChIP-seq kit for TFs (Diagenode, C01010055) with modifications of the manual detailed below. Lysed samples were sonicated using the E220 sonicator (Covaris, 500239) in microTUBE AFA Fiber Pre-Slit Snap-Cap tubes (Covaris, 520045) with settings for 200-bp DNA shearing. Sheared chromatin was immunoprecipitated with 2.5 µg

HA antibody (CST, HA-Tag (C29F4) Rabbit mAb #3724), 2.5 µg H3K27ac antibody (Diagenode, C15410196) or 2.5 µg IgG antibody (Diagenode, C15410206, RRID AB_2722554). Eluted and decross-linked DNA was purified with MicroChIP DiaPure columns (Diagenode, C03040001) and eluted in 30 µl of nuclease-free water. ChIP and input libraries for sequencing were prepared with ThruPLEX DNA-Seq kit (Takara, R400674) and DNA Single Index kit, 12S Set A (Takara, R400695). Size selection steps were performed with Magbio Genomics HighPrep PCR beads (Fisher Scientific, 50-165-6582). The libraries were sequenced using the Illumina NextSeq 500 platform and the 150-bp paired-end configuration to obtain at least 20 million reads per sample.

Annexin V apoptosis staining

HNT-34-dTAG cells were treated with 500nM $dTAG^V$-1 or DMSO in culture for three days and then collected. Cells were washed twice with cold PBS with 2% FBS and then resuspended in Annexin V Binding Buffer (Cat. No. 422201) at a concentration of $1x10^6$ cells/ml. 100 µl of cell suspension was transferred to an Eppendorf tube and mixed with 5 µl of APC Annexin V. Propidium iodide was added then cells were gently vortexed and incubated for 15 min at room temperature (25°C), in the dark. 400 µl of Annexin V Binding Buffer was then added to each sample and cells were analyzed by flow cytometry.

CRISPR engineering of cell lines and primary AML cells

Cell lines were electroporated using the Lonza 4D Nucleofector with 20 µl Nucleocuvette strips as described[1,10]. Cas9-sgRNA ribonucleoprotein complexes (RNPs) were made by combining 50 pmol of Cas9 protein (IDT) and 100 pmol of chemically synthesized sgRNA (Synthego) targeting the C-terminus of *MECOM* and incubating at 21 °C for 15 min. Between 2×10^5 and 5×10^5 cells were resuspended in 20 µl P3 solution, mixed with RNP and underwent nucleofection with program EO-100. Cells were returned to appropriate cell culture medium and supplemented with 50µl of crude rAAV HDR donor (see methods) and 1µM of alt-R HDR enhancer (IDT, 10007910). 24 hours later, cells were washed once with 1x PBS and replated in fresh cell culture medium. 72 hr after electroporation cells were analyzed via flow cytometry to assess GFP expression. Most AML models we chose, including MUTZ-3, UCSD-AML1, and HNT-34, exhibit a translocation or inversion on chromosome 3[11,12] which hyperactivates expression of a single copy of the *MECOM* locus. This allowed for sorting of polygenic populations of GFP^+ cells in which the transactivated *MECOM* allele was correctly tagged with the $FKBP12^{F36V}$ degron cassette. On-target editing and successful knock-in was further confirmed by genomic DNA PCR. Genomic DNA was extracted using the DNeasy kit (QIAGEN) according to the manufacturer's instructions. PCR was performed using Platinum II Hotstart Mastermix (Thermo Fisher Scientific) and primers flanking the repair site. For OCI-AML4 cells, which do not have an inversion or translocation on chromosome 3 but still exhibit relatively high MECOM expression, following CRISPR editing single cells were plated by limiting dilution in 96-well plates and clonally expanded. After 2 weeks of expansion, genomic DNA was extracted from clones and screened via PCR for biallelic tagging. A homozygous clone was identified and further expanded in culture for experimental use.

Primary AML cells were electroporated using the Lonza 4D Nucleofector with 20 µl Nucleocuvette strips. For Cas9 nuclease experiments, 50 pmol of Cas9 protein was mixed with a total 100 pmol of sgRNAs targeting either the *MECOM* or *AAVS1* loci alone or with sgRNAs targeting the CEBPA cisRE. For CRISPRa mRNA experiments, 2.5 µg of dCas9-VPR mRNA was mixed with 100 pmol of sgRNAs targeting the CEBPA cisRE or a non-targeting control. In all experiments between 2×10^5 and 5×10^5 cells were resuspended in 20 µl P3 solution, mixed with the corresponding CRISPR reagents, and underwent nucleofection with program DZ-100. Electroporated cells were maintained in MS-5 co-culture with the appropriate medium. To assess editing efficiencies in Cas9 nuclease experiments, genomic DNA PCR was performed using Platinum II Hotstart Mastermix (Thermo Fisher Scientific) and edited allele frequency was detected by Sanger sequencing and analyzed by ICE[13] (**Table S1-2**). The effect of CRISPR editing on gene expression was assessed by RT-qPCR three days after electroporation.

Recombinant AAV production

The triple-transfection method was used to generate crude rAAV lysates[14]. HEK293T cells were plated in a 10 cm dish in 1X Penicillin-streptomycin-Glutamine and 10% FBS in DMEM. At 80% confluence, the medium was replaced with fresh medium. Then, cells were triple transfected using polyethylenimine Max (PEI Max, Polysciences 24765-1) with 12 µg of pAAVhelper, 7.5 µg of pRep2Cap6, and 7.5 µg of transfer plasmid (PEI:DNA = 3:1), in DMEM without phenol red (Life Technologies 31053036). 3 days after transfection, cells were scraped and collected by spinning at 1300 RPM for 5 min. Cell pellet was resuspended in 1-2 mL DPBS without calcium or magnesium and lysed by three rounds of freeze-thaw. This was accomplished by placing them alternately in a dry ice/ethanol bath until completely frozen and in a water bath of 37 °C until completely thawed. After the final thaw, cell lysate was spun at 1300 RPM for 5 min and the supernatant was filtered through a 0.22 µm syringe filter to yield a crude viral lysate. Viral lysates were stored in 25 µL aliquots at 4C for up to 4 weeks, and at -80C thereafter.

RNA Isolation, Reverse Transcription and Real-time PCR

RNA was harvested by the RNeasy Micro kit (QIAGEN, 74004) and quantified by nanodrop. 1 microgram of total RNA was then reverse transcribed using iScript cDNA synthesis kit following manufacturer's instructions. The reverse transcribed cDNA was then diluted (1:20) and real time PCR was run using Biorad iQ SYBR green supermix. Data was normalized by loading control (*ACTB*) and presented as fold change compared to control samples using the delta-delta CT (ΔΔCT) method.

Quantitative Mass Spectrometry-Based Proteomics

MUTZ-3 cells were treated with DMSO or 500 nM dTAGV-1 and lysed in a buffer containing 8 M urea and 200 mM EPPS at pH 8.5 with protease inhibitors. The lysates were generated using a probe sonicator (20 pulses of 0.5 seconds at level 3). Protein concentration was measured using a BCA assay, and 50 µg of protein was aliquoted for each condition. Proteins were reduced with TCEP for 15 minutes at room temperature (RT) and alkylated with 10 mM iodoacetamide for 30

minutes in the dark at RT. Precipitation was performed using chloroform/methanol, as described previously[15]. Samples were digested overnight with LysC and trypsin (1:100 enzyme/protein ratio) at 37°C on a ThermoMixer set to 1,200 rpm. After digestion, peptides were labeled with TMTpro 18-plex reagents (1:2 peptide/reagent mass ratio) for 1 hour with constant shaking at 1,200 rpm. Excess TMT reagent was quenched with 0.3% hydroxylamine for 15 minutes at RT. The samples were mixed in equal proportions across all TMT channels, pooled, and dried using a Speedvac. The pooled peptides were desalted with a 100-mg Sep-Pak solid-phase extraction cartridge. After desalting, the peptides were dried, resuspended in a buffer (10 mM ammonium bicarbonate, 5% acetonitrile, pH 8.0), and fractionated into a 96-well plate using basic pH reversed-phase HPLC with an Agilent 300 Extend-C18 column. Fractionation was performed with a 50-minute linear gradient of 13–43% buffer (10 mM ammonium bicarbonate, 90% acetonitrile, pH 8.0) at a flow rate of 0.25 mL/min. The peptide mixture was combined into 24 fractions and were desalted using StageTips[15]. Forty percent of the resuspended sample (10 µL of 5% acetonitrile, 5% FA) was analyzed on an Orbitrap Eclipse using a high-resolution MS2-based method.

Liquid chromatography and mass spectrometry data acquisition.

Mass spectrometry data were collected using a Orbitrap Eclipse mass spectrometer (Thermo Fisher Scientific, San Jose, CA) coupled with Neo Vanquish liquid chromatograph. Peptides were separated on a 100 µm inner diameter microcapillary column packed with ～35cm of Accucore C18 resin (2.6 µm, 150 Å, Thermo Fisher Scientific). For each analysis, we loaded ~2 µg onto the column. Peptides were separated using a 90 min gradient of 5 to 29% acetonitrile in 0.125% formic acid with a flow rate of 400 nL/min. The scan sequence began with an Orbitrap MS^1 spectrum with the following parameters: resolution 60K, scan range 350-1350, automatic gain control (AGC) target 100%, maximum injection time “auto,” and centroid spectrum data type. We use a cycle time of 1s for MS^2 analysis which consisted of HCD high-energy collision dissociation with the following parameters: resolution 50K, AGC 200%, maximum injection time 86ms, isolation window 0.6 Th, normalized collision energy (NCE) 36%, and centroid spectrum data type. Dynamic exclusion was set to automatic. The FAIMS compensation voltages (CV) were -40, -60, and -80V.

CRISPR library and individual sgRNA cloning

The following protocol was used for creating a sgRNA lentiviral library or individual sgRNA lentiviruses. The sgRNA library for both screens was designed to target a conserved network of MECOM-regulated cisREs. We first mined the ENCODE Consortium’s recently published repository of functionally validated sgRNA sequences[16] that overlapped our genomic regions of interest and selected 5 sgRNA sequences per region. For regions absent from the ENCODE database or corresponding to less than 5 validated sgRNAs we utilized the CRISPick tool from The Broad Institute to design additional sgRNAs. Oligonucleotide pools for CRISPR screens were ordered from IDT at a 50 pmol scale (standard desalting) and resuspended at a 10 µM concentration **(Table S8)**. Single oligonucleotides for individual sgRNA lentiviruses were ordered from Azenta Life Sciences at a 25 nmol scale (standard desalting) and resuspended at a 10 µM concentration.

An initial extension reaction was performed using the oligo pool **(Table S8)** or individual oligonucleotides, NEB Q5 Hot Start High-Fidelity 2X Master Mix (M0492L) and extension primers. The following parameters were used for extension: 98ºC for 2 minutes; 10 cycles of (64ºC for 30 seconds and 72ºC for 20 seconds); 72ºC for 2 minutes; and hold at 4ºC. The product was purified using the Monarch® PCR & DNA Cleanup Kit (NEB, T1030), and eluted in 25 mL of water. All sgRNAs were cloned into a modified CROP-seq-opti vector[17] in which the the puromycin resistance cassette was replaced with tag red fluorescent protein (tag-RFP) to facilitate lentiviral titration and precise FACS-enrichment of infected cells. BsiWI (NEB) and MluI (NEB) were used to excise the puromycin resistance marker and a gBlock with the tag-RFP sequence was cloned into the digested vector using the same restriction site overhangs. This modified vector was digested at 37ºC for 1 hour and purified with Monarch® DNA Gel Extraction Kit (NEB #T1020).

An NEBuilder HiFi DNA Assembly (NEB, #E2621) reaction was performed using 500 fmol of vector and 10,000 fmol of purified extension reaction product (with the volume required for each calculated using its fragment length and its concentration measured by Nanodrop) with 10 µl of 2x NEBuilder HiFi DNA Assembly Master Mix and nuclease-free water to a final reaction volume of 20 µl. The reaction was incubated at 37ºC for 2 hours. For library cloning, 5 µl of crude NEBuilder HiFi DNA Assembly product were transformed into Endura Electrocompetent Cells (Biosearch Technologies, 71003-038) using the Biorad Gene Pulser Xcell Total Electroporation System (1652660) with the following parameters: 1.8 kV, 25 µF and 200 U. Bacteria were recovered for 20 minutes in the kit's recovery media. 2 µL of bacteria were used to create 4 serial dilutions to evaluate the transformation efficiency (and ensure at least 100x coverage of the library) and the remaining bacteria were inoculated in 500 mL of LB with 100 µg/ml of ampicillin and grown overnight at 30ºC. 16-18 hours later, plasmid DNA was extracted using the NucleoBond Xtra Maxi kit for endotoxin-free plasmid DNA (Macherey-Nagel, 740424.50) and eluted in 400 µL of nuclease-free water. For single sgRNA cloning, 2 uL of crude NEBuilder HiFi DNA Assembly product were transformed into chemically competent NEB® 10-beta Competent *E. coli* per the manufacturer's recommended protocol and plated onto LB agar plates with 100 µg/ml of ampicillin and grown overnight at 37ºC. The following day, colonies were picked, expanded overnight at 37ºC, and plasmid DNA was extracted using the Monarch® Plasmid Miniprep Kit and eluted in 30 µL of nuclease-free water. Plasmid DNA was then sequenced by whole plasmid sequencing (Primordium) to identify positive clones.

MECOM lentiviral overexpression constructs

The EVI1 isoform of the *MECOM* locus was used for all lentiviral overexpression experiments. A construct containing the EVI1 coding sequence and IRES-eGFP cassette placed downstream from a constitutive promoter (HIV/MSCV hybrid LTR)[18] were obtained from Voit et al. 2023[19]. For dTAG^{V}-1 rescue experiments, the IRES-eGFP cassette was first replaced with IRES-TagRFP using EcoRI and PacI restriction sites. The PLASS mutation was introduced into the PLDLS motif of the EVI1 coding sequence using two PstI restriction sites flanking the PLDLS motif. This small fragment was excised and replaced with an identical sequence, except for the PLDLS motif, which was altered to PLASS.

CRISPRi screen

MUTZ-3-dTAG cells were transduced at high MOI with separate lentiviruses packaged with TRE-KRAB-dCas9-IRES-BFP[20] and pLVX-EF1alpha-Tet3G (Takara #631359). TRE-KRAB-dCas9-IRES-BFP was a gift from Eric Lander (Addgene plasmid # 85449; http://n2t.net/addgene:85449 ; RRID:Addgene_85449). The next day, to bypass G418 selection and select for co-transduced cells, 1µg/ml of doxycycline was added and 48 hours later BFP^+ cells were sorted by FACS. These MUTZ-3 cells stabling expressing inducible dCas9-KRAB were then transduced with a MECOM-regulated cisRE-targeting lentiviral sgRNA library at a low MOI (~0.33) in technical triplicate at ~2000x coverage (cells/sgRNA). 24 hours after transduction, 1µg/ml doxycycline was added to induce dCas9-KRAB expression and 48 hours after transduction 500nM of $dTAG^V$-1 was added to all replicates. Cells were maintained in culture with regular media changes and supplementation of fresh doxycycline and $dTAG^V$-1 every three days. A population of cells not treated with $dTAG^V$-1 or not transduced with the sgRNA library were also maintained as controls to ensure $dTAG^V$-1 treatment induced robust MUTZ-3 differentiation and that expression of the sgRNA library resulted in a relative enrichment of $CD34^+$ stem-like cells, respectively **(Fig. S5A)**. 14 days post-transduction, residual $CD34^+$ stem-like cells were sorted by FACS and genomic DNA was extracted with the DNeasy kit (QIAGEN) according to the manufacturer's instructions. PCR on genomic DNA from all replicates was performed using Titanium Taq DNA Polymerase and PCR buffer (Clontech Takara Cat# 639208) according to The Broad Institute Genetic Perturbation Platform's protocol for PCR of sgRNAs from genomic DNA for Illumina sequencing (https://portals.broadinstitute.org/gpp/public/resources/protocols). In brief, barcoded P5 and P7 PCR primers (table) were used to amplify the sgRNA spacer sequence from the genomically-integrated lentiviral sgRNA expression cassette. An aliquot of sgRNA library plasmid DNA was also used as a template for this PCR to assess the coverage of the cloned sgRNA library and generate a baseline sgRNA distribution to assess relative enrichment or depletion in our screens. Amplicon libraries were purified with AMPure XP-PCR magnetic beads, and pooled at an equimolar concentration. Libraries were subject to paired-end sequencing using NextSeq550 High Output kits with 75 cycles to ensure at least 20 million reads per library.

CRISPRa screen

MUTZ-3-dTAG cells were transduced at a high MOI with lentivirus packaged with pXPR_120 (dCas9-VPR-2A-BlastR)[21]. pXPR_120 was a gift from John Doench & David Root (Addgene plasmid # 96917; http://n2t.net/addgene:96917; RRID: Addgene_96917). 48 hours after transduction, cells were treated with 10 µg/ml of Blasticidin for 7 days to select for cells stably and constitutively expressing dCas9-VPR. These MUTZ-3 cells were then transduced with a MECOM-regulated cisRE-targeting lentiviral sgRNA library at a low MOI (~0.33) in technical triplicate at ~2000x coverage (cells/sgRNA). Cells were maintained in culture with regular media changes every three days. 14 days post-transduction, cells in the bottom 5% of CD34 expression measured via flow-cytometry were sorted by FACS. Genomic DNA extraction, library preparation, and sequencing of CRISPRa libraries was all performed as previously described for the CRISPRi screen.

In vitro transcription of dCas9-VPR mRNA

An in vitro transcription template encoding dSpCas9-VPR was a gift from Rasmus Bak (Addgene plasmid # 205247 ; http://n2t.net/addgene:205247 ; RRID:Addgene_205247)[22]. The plasmid was digested with SapI (NEB), a restriction site immediately downstream from the encoded polyA tail, for 1 hr at 37ºC and the linear transcription template was purified using Monarch® DNA Gel Extraction Kit and eluted in 20 uL of nuclease free water. dCas9-VPR mRNA was transcribed from this template using the HiScribe T7 High Yield RNA Synthesis Kit (New England Biolabs) according to the manufacturer's recommended protocol for high-yield synthesis with the following changes: UTP was fully replaced with N1-methylpseudouridine-5′-triphosphate (TriLink Biotechnologies) and co-transcriptional capping by CleanCap Reagent AG (TriLink Biotechnologies) was used at a ratio of 4:1 with GTP. mRNA products were precipitated in 2.5 M lithium chloride, washed twice with 70% ethanol, dissolved in nuclease-free water, and stored at −80 °C.

AML cell line bulk RNA-seq analysis

FASTQ files were demultiplexed with bcl2fastq and aligned to the hg38 reference genome using bowtie2 (version 2.5.2). Sam files were sorted, indexed, and converted to bam files with Samtools (version 1.18). For data visualization in a genome browser, bam files were converted to bigwig files using the bamCoverage package from deepTools (version 3.5.4) normalizing by counts per million (CPM). Count tables for genes were also generated from bam files using the featureCounts package from Subread (version 2.0.6) and differential gene expression analysis was performed using DESeq2 (version 1.40.2). Results were visualized using ggplot2 (version 3.4.4).

AML cell line ATAC-seq analysis

FASTQ files were demultiplexed with bcl2fastq and aligned to the hg38 reference genome using bowtie2 (version 2.5.2). Sam files were sorted, indexed, and converted to bam files with Samtools (version 1.18). For data visualization in a genome browser, bam files were converted to bigwig files using the bamCoverage package from deepTools (version 3.5.4) normalizing by counts per million (CPM). Peak calling was performed using MACS2 (version 2.2.9.1) with the flags --shift -100 and --extsize 200 to generate narrowPeak files. NarrowPeak files from DMSO and dTAG^{V}-1-treated cells were then merged and converted to SAF format to generate a consensus peak set for each cell line. This consensus peak file and corresponding bam files were then processed using the featureCounts package from Subread (version 2.0.6) to generate a count table for ATAC peaks. Differential peak accessibility analysis was performed using DESeq2 (version 1.40.2). Results were visualized using ggplot2 (version 3.4.4). Transcription factor binding motif enrichment analysis was performed using Analysis of Motif Enrichment (AME) from The MEME Suite (version 5.5.0) to assess enrichment of motifs from the JASPAR CORE (2022) Vertebrates Non-Redundant database[23].

PRO-seq analysis

All custom scripts described herein are available on the AdelmanLab GitHub (https://github.com/AdelmanLab/NIH_scripts). Using a custom script (trim_and_filter_PE.pl), FASTQ read pairs were trimmed to 41bp per mate, and read pairs with a minimum average base quality score of 20 were retained. Read pairs were further trimmed using cutadapt (version 4.1) to remove adapter sequences and low-quality 3' bases (–match-read-wildcards -m 20 -q 10). R1 reads, corresponding to RNA 3' ends, were then aligned to the spiked in Drosophila genome index (dm6) using BWA, with those reads not mapping to the spike genome serving as input to the primary genome alignment step. Reads mapping to the hg38 reference genome were then sorted, via samtools (version 1.3.1 -n), and subsequently converted to bam files. The bam files are converted to bigwig files by bamCoverage of deepTools (version 3.5). For metagene plots, bigwig files of three replicates of each group and combined and averaged using WiggleTools.

ChIP-seq analysis

For HA and H3K27ac ChIP-seq experiments, FASTQ files were demultiplexed with bcl2fastq and aligned to the hg38 reference genome using bowtie2 (version 2.5.2). Sam files were sorted, indexed and converted to bam files with Samtools (version 1.18). For data visualization in a genome browser, bam files were converted to bigwig files using the bamCoverage package from deepTools (version 3.5.4) normalizing by counts per million (CPM). Peak calling was performed using MACS2 (version 2.2.9.1). NarrowPeak files from DMSO and dTAG^V-1-treated cells were then merged and converted to SAF format to generate a consensus peak set for each cell line. This consensus peak file and corresponding bam files were then processed using the featureCounts package from Subread (version 2.0.6) to generate a count table for ChIP peaks. CtBP2 ChIP-seq summary data (Bigwig files aligned to hg19) were downloaded from GSE236010 and converted to Bigwig files aligned to hg38 using the liftOver tool from UCSC. Bigwig files were analyzed with the computeMatrix and plotHeatmap packages from deepTools to assess the deposition of H3K27ac signal and association of CtBP2 at MECOM-regulated cisREs.

Identification of MECOM-regulated *cis*-regulatory elements

Using external parental MUTZ-3 MECOM ChIP-seq data[24], we performed peak calling using MACS2 as described above. We intersected the genomic coordinates of the ChIP-seq peaks with our ATAC-seq peak calls to identify MECOM-bound sites with open chromatin. To enrich for a set of sites with both strong MECOM-binding and a change in chromatin accessibility following MECOM degradation we filtered our overlapping sites using the following parameters: ATAC-seq (6hr dTAG^V-1 vs DMSO) DESeq2 p-value <0.01 and MECOM ChIP-seq MACS2 peak Pscore>50 (PScore=-log10pvalue*10). This analysis resulted in 837 genomic intervals (MECOM cisRE network).

Gene set enrichment analysis

We used GSEApy[25] (https://github.com/zqfang/GSEApy) for all GSEA analyses to determine the enrichment of genes and cisREs under direct regulation of MECOM as determined by our MUTZ-

3 dTAG studies in other *MECOM-FKBP12^{F36V}* cell line models. Significant enrichment of the gene and cisRE sets was determined using GSEAPrerank in which the RNA-seq and ATAC-seq data from UCSD-AML1, HNT-34, and OCI-AML4 cells treated with dTAG^{V}-1 vs. DMSO were pre-ranked by $\log_2$ fold change. For enrichment analyses of MECOM-regulated cisREs, the genomic coordinates of ATAC-seq peaks were used in place of gene name. ATAC-seq peaks from all datasets were overlapped with the MUTZ-3 consensus cisRE network using the intersect package from bedtools (version 2.31.0) and renamed to the same genomic coordinates of the corresponding MUTZ-3 peak. GSEA was performed using 1,000 permutations to determine significance.

Genome Regions Enrichment of Annotations Tool (GREAT) analysis

Differentially accessible ATAC-peaks that also overlapped with MECOM ChIP-seq peaks (837 site cisRE network) **(Fig. 2E)** were linked to genes based on proximity using Genome Regions Enrichment of Annotations Tool (GREAT; version 4.0.4). Each cisRE was associated to genomic loci using the “basal plus extension” mode with the following parameters: proximal 5kB upstream, 1kb downstream, plus distal up to 1000kb.

Mass spectrometry data analysis

Mass spectrometry data were analyzed using the open-source Comet algorithm (release_2019010), following a previously established pipeline and a customized FASTA-formatted database[26–29]. This database included common contaminants and reversed sequences (Uniprot Human, 2021). The search parameters were set as follows: 50 PPM precursor tolerance, fully tryptic peptides, 0.02 Da fragment ion tolerance, and static modifications of TMTpro18 (+304.2071 Da) on lysine residues and peptide N-termini, as well as carbamidomethylation of cysteine residues (+57.0214 Da). Oxidation of methionine residues (+15.9949 Da) was included as a variable modification.

Peptide spectral matches were filtered to maintain a false discovery rate (FDR) of <1% using linear discriminant analysis with a target-decoy strategy. Further filtering ensured a protein-level FDR of 1% across the dataset, and proteins were grouped accordingly. Reporter ion intensities were corrected for TMT reagent impurities following the manufacturer's specifications. MS2 spectra required a total signal-to-noise (S/N) sum of at least 180 across all reporter ions for quantification. For proteins, S/N measurements of corresponding peptides were summed and normalized to ensure consistent loading across all channels. Finally, protein abundance measurements were scaled such that the total summed S/N for each protein across all channels was set to 100, providing relative abundance measurements.

CRISPR screen analysis

Quality control and enrichment analysis of CRISPR screen sequencing data was performed using MAGeCKFlute pipeline[30] (version 0.5.9.5). Briefly, FASTQ files were mapped using the count function with the control norm-method. Enrichment of cisRE-targeting sgRNAs was calculated

using the test function comparing either the sorted CD34$^+$ population (CRISPRi screen) or the sorted CD34-low population (CRISPRa screen) to the plasmid DNA library, with the MAGeCK Robust Rank Algorithm (RRA) using non-targeting and AAVS1-targeting sgRNAs as negative controls.

Single cell RNA-seq analysis:

Filtered count matrices were downloaded from GEO (GSE235063) and analyzed according to Lambo et al. 2023[3]. Briefly, data were log normalized to 10,000 counts and scaled using Seurat[31]. Dimensionality reduction was performed by Uniform Manifold Approximation and Projection (UMAP) using the uwot package (version 0.1.1) after initialization using principal component analysis (PCA). Calculation of nearest neighbors (nn) was performed by applying the annoy algorithm. Inferred cell type, malignancy and other metadata were carried over from Lambo et al 2023[3].

Malignant cells were selected according to their annotation. Signature scores were calculated using the Addmodulescore function in Seurat[31] using published signatures of HSC and monocyte populations from Lambo et al. 2023[3] and MECOM-regulated genes from this study **(Table S7).** Comparison of signature scores between MECOM positive and MECOM negative samples was performed using a Wilcoxon signed rank test adjusted for multiple testing correction using Benjamini Hochberg (BH) correction. Differential expression was calculated using MAST[32] (version 1.16) as implemented within the findMarker feature in Seurat. Comparisons were performed by randomly taking the average over ten iterations of 1,000 randomly sampled cells from both samples expressing MECOM and samples not expressing MECOM to avoid uninformative p- values close to zero.

Primary AML bulk RNA-seq analysis

Data were downloaded and processed as described in Lambo et al. 2023[3] Samples were deemed MECOM positive if expression was over 32 transcripts per million. Gene set enrichment was calculated using gene set variation analysis (GSVA v1.38.2)[33] using an HSC signature derived from Lambo at al. 2023.

Single cell ATAC-seq analysis:

Filtered fragment files were downloaded from GEO (GSE235308) and remission samples were analyzed according to Lambo et al. 2023[3]. Briefly, cells were clustered using iterative latent semantic indexing (LSI)[34] and annotations, including cell type labels, malignancy status, peak calling, and linked scRNA cells were transferred from the original publication. Linkage between scRNA profiles and scATAC profiles was also based on this metadata and were originally identified using Seurat findTransferAnchor[31]. Markov Affinity-based Graph Imputation of Cells (MAGIC; version 2.0)[35] was used to impute weights based on identified nearest neighbors in the dimensionality reduction. Motif analysis was performed using Chromvar[36] using annotations derived from cisBP[37].

Subsequently, MECOM cisRE insertion scores were calculated by summing up insertions within peaks identified six hours post dTAG^{V}-1 treatment **(Table S6)** and normalized by the total insertions in promoter regions. This was performed to correct for sequencing depth and differences in signal to noise ratio within cells. Lineages scores were defined using lineage defining peaks, which were derived from Lambo et al 2023[3]. Lineage scores were calculated for each cell separately by combining the total insertions within lineage defining peaks of each separate lineage (Myeloid, Lymphoid, Erythroid) and dividing this number by the total insertions of lineage defining peaks from the other lineages. Correlations between scores were calculated using Spearman correlation.

Trajectory analysis was performed using Monocle (version 3.0)[38] between clusters defined by Seurat findClusters[31]. Trajectories were drawn between the cluster with the highest number of identified HSCs[31], and CD34 positive cells and the cluster with the highest number of monocytes and CD14 positive cells. Cells along the cluster were binned in 100 bins of equal size and profiles were aggregated, z-score normalized and smoothened using a rolling mean across the trajectory. A heatmap of cluster scores was calculated using the mean score of each cluster and scaled using z-score normalization.

QUANTIFICATION AND STATISTICAL ANALYSIS

Statistical tests and statistical significance are indicated in the figure legends. All error bars represent standard error of the mean unless otherwise indicated.

SUPPLEMENTAL FIGURE LEGENDS

Figure S1. FKBP12^{F36V} degron enables targeted MECOM degradation in additional AML cell lines

(A) Time course western blot analysis of MECOM protein levels in UCSD-AML1 MECOM-FKBP12^{F36V} and HNT-34 MECOM-FKBP12^{F36V} cells treated with 500nM dTAG^{V}-1 vs. DMSO.

(B-C) Histogram of CD34 expression in UCSD-AML1 MECOM-FKBP12^{F36V} and WT UCSD-AML1 cells 6 days after treatment with 500nM dTAG^{V}-1 vs. DMSO. n = 3 independent replicates, mean and SEM are shown. Two-sided Student t test was used for comparison. *$p < 0.05$, ns, not significant.

(D) Confocal microscopy images of UCSD-AML1 MECOM-FKBP12^{F36V} cells 9 days after treatment with 500nM dTAG^{V}-1 vs. DMSO following cytospin and May-Grünwald Giemsa staining.

(E) Histogram showing Annexin-V staining of HNT-34 MECOM-FKBP12^{F36V} cells 3 days after treatment with 500nM dTAG^{V}-1 vs. DMSO. n = 3 independent replicates.

(F) Viable cell count by trypan blue exclusion of HNT-34 MECOM-FKBP12^{F36V} 6 days after treatment with 500nM dTAG^{V}-1 vs. DMSO. n = 3 independent replicates, mean and SEM are shown. Two-sided Student t test was used for comparison. ****$p < 0.0001$.

(G-H) Cell cycle analysis of CD34^{+} MUTZ-3 MECOM-FKBP12^{F36V} and UCSD-AML1 MECOM-FKBP12^{F36V} cells after treatment with 500nM dTAG^{V}-1 vs. DMSO. **(G)** 48 hours post-treatment, cells were incubated with 10uM EdU for 2 hours then processed for flow cytometry analysis with PI staining. **(H)** Stacked bar plot comparing differences in cell cycle populations (G0/1, G2, S) of dTAG^{V}-1 vs. DMSO treated samples. n = 3 independent replicates, mean and SEM are shown. Two-sided Student t test was used for comparison. ****$p < 0.0001$.

Figure S2. MECOM-regulated gene and chromatin networks are conserved in UCSD-AML1 and HNT-34 cell line models

(A-B) Volcano plot and heatmap representing changes in gene expression assessed via bulk RNA-seq of MUTZ-3 dTAG cells treated with dTAG^{V}-1 or DMSO for 6 hours (n=3). MECOM down genes[19] are highlighted in blue data points.

(C-D) Volcano plot and heatmap representing changes in gene expression assessed via bulk RNA-seq of MUTZ-3 dTAG cells treated with dTAG^{V}-1 or DMSO for 24 hours (n=3). MECOM down genes are highlighted in blue data points.

(E-H) Volcano plots showing changes in gene expression assessed via bulk RNA-seq of UCSD-AML1 MECOM-FKBP12^{F36V} and HNT-34 MECOM-FKBP12^{F36V} cells treated with dTAG^{V}-1 or DMSO for 6 hours (n=3) and gene set enrichment analysis (GSEA) compared to MECOM network genes. MECOM network genes as identified from MUTZ-3 experiments in **Fig. 2** are highlighted in red data points.

(I-L) Volcano plots showing changes in chromatin accessibility assessed via ATAC-seq of UCSD-AML1 MECOM-FKBP12^{F36V} and HNT-34 MECOM-FKBP12^{F36V} cells treated with dTAG^{V}-1 or DMSO for 6 hours (n=3) and gene set enrichment analysis (GSEA) of ATAC-peaks compared to MECOM network cisREs. MECOM network cisREs as identified from MUTZ-3 experiments in **Fig. 2** are highlighted in red data points.

Figure S3. Primary MLL-rearranged AML cohort is stratified by MECOM expression status

(A) Representation of patient samples at diagnosis sequenced using bulk RNA-seq as part of the AAML1031 trial (n=701) (Aplenc et al 2020[39], Lambo et al 2023[3]). (Left) From inside to outside, the inner circle shows a broad classification used in Lambo et al 2023[3], the second ring shows a more detailed cytogenetic classification of the MLLr subgroup and other AMLs and the outer ring shows whether MECOM was found to be expressed (log_2 expression > 5). (Right) Graph showing the same classification of all samples having MECOM expression. Only samples with a blast cell content over 40% were included.

(B) Expression of MECOM across the MLLr leukemias included in the AAML1031 cohort. x-axis represents log_2 transformed expression value, y-axis represents the kernel density.

(C) Kaplan meier survival curves showing five-year OS and five-year EFS split by MLLr leukemias expressing MECOM (n=49) and MLLr leukemias not expressing MECOM (n=126).

(D) Bar chart showing the expression of HSC-associated genes CD34 and SPINK2 in samples stratified by MECOM expression. BH-adjusted p-values were calculated using two-sided Wilcoxon signed-rank tests.

(E) Heatmap showing the expression pattern of HSC signature genes derived from normal HSCs (Lambo et al 2023[3]) across 175 MLLr samples within the AAML1031 cohort. Signature scores were calculated using GSVA.

(F) Scatter plot showing the correlation between HSC signature scores derived using GSVA and the log_2 expression of MECOM.

(G) Violin plots showing the expression of MECOM across individual cells of 11 MLLr samples included in the scRNA cohort of the AAML1031 trial. Cells were divided by non-malignant as described in (Lambo et al 2023[3]), samples taken at diagnosis and at relapse. BH adjusted p-values were calculated using two-sided Wilcoxon signed-rank tests.

Figure S4. MECOM-regulated gene and chromatin networks are conserved in MLL-rearranged, MECOM^{+} OCI-AML4 cells

(A) Depiction of myeloid cell lines from The Cancer Dependency Map and their relative MECOM expression.

(B) Genomic DNA PCR strategy to screen for MECOM-FKBP12^{F36V} biallelically-tagged OCI-AML4 isogenic clones. OCI-AML4 cells lack an activating translocation or rearrangement at the *MECOM* locus, thus requiring both *MECOM* alleles to be tagged with an FKBP12^{F36V} degron. PCR primers flanking the C-terminus of *MECOM* were used to identify a biallelically-tagged, isogenic clone (outlined in red).

(C) Time course western blot analysis of MECOM protein levels in OCI-AML4 MECOM-FKBP12^{F36V} clone treated with 500nM dTAG^{V}-1 vs. DMSO.

(D) Volcano plot representing changes in gene expression assessed via RNA-seq of OCI-AML4 MECOM-FKBP12^{F36V} clone treated with dTAG^{V}-1 vs. DMSO for 6 hours (n=3). MECOM network genes as identified from MUTZ-3 experiments in **Fig. 2** are highlighted in red data points.

(E) Heatmaps displaying differential expression of individual MECOM network genes in DMSO and dTAG^{V}-1 conditions from experiments in **Fig. S4D**.

(F) Gene set enrichment analysis (GSEA) of differentially expressed genes from experiments in **Fig. S4D** compared to MECOM network genes.

(G) Volcano plot representing changes in chromatin accessibility assessed via ATAC-seq of OCI-AML4 MECOM-FKBP12^{F36V} clone treated with dTAG^{V}-1 vs. DMSO for 6 hours (n=3). MECOM-regulated *cis*-regulatory elements as identified from MUTZ-3 experiments in **Fig. 2** are highlighted in red.

(H) Heatmap displaying differential accessibility of individual MECOM-regulated *cis*-regulatory elements in DMSO vs. dTAG^{V}-1 samples from experiments in **Fig. S4G**.

(I) Gene set enrichment analysis (GSEA) of differentially accessible ATAC-peaks from experiments in **Fig. S4G** compared to MECOM-regulated *cis*-regulatory elements.

Figure S5. CEBPA cisRE function is conserved in other AML cells

(A) Flow cytometry plot from MUTZ-3 CRISPRi screen 14 days in culture. The no sgRNA library condition compared to sgRNA library transduced condition demonstrates the relative enrichment of the phenotypically rescued, CD34^{+} cells in sgRNA library expressing cells.

(B) Orthogonal validation of CRISPRi screen in UCSD-AML1 cells. UCSD-AML1 MECOM-FKBP12^{F36V} dCas9-KRAB cells were infected with sgRNA-expressing lentiviruses targeting either the CEBPA cisRE or a non-targeting (NT) sequence. 48 hours after transduction, cells were treated with 500nM dTAG^{V}-1 vs. DMSO. (Left) Histogram shows CD34 expression at day 9. (Right) Percentage of CD34^{+} cells at day 9. n = 3 independent replicates, mean and SEM are shown. Two-sided Student t test was used for comparison. ****$p < 0.0001$.

(C) Orthogonal validation of CRISPRa screen in UCSD-AML1 cells. UCSD-AML1 dCas9-VPR cells were infected with sgRNA-expressing lentiviruses targeting either the CEBPA cisRE or a non-targeting (NT) sequence. (Left) Histogram shows CD34 expression at day 9. (Right) Percentage of $CD34^{+}$ cells at day 9. n = 3 independent replicates, mean and SEM are shown. Two-sided Student t test was used for comparison. *p < 0.05, ns, not significant.

(D) UMAP showing a trajectory inferred using Monocle from inferred HSCs to inferred Monocytes (see also **Figure 4**).

(E) Seurat identified clusters in the scATAC data. Each color represents a different cluster.

(F) Expression of *MECOM* and *CEBPA* from scaled counts derived from linked scRNA data across cells from remissions.

(G) UMAP showing the scaled TF motif enrichment scores (ChromVAR deviation scores) of MECOM and CEBPA motifs across all remission cells.

(H) Heatmaps showing the scaled expression of identified genes (n=122) and cisREs (n=837) directly regulated by MECOM **(Figure 2)** along the pseudotime shown in **(E)**. Each column represents an aggregated minibulk from cells across the inferred pseudotime (100 bins total). Gene expression scores are counts derived from linked scRNA samples, peak insertions were normalized by TSS insertions and Tn5 bias. Both gene expression and chromatin accessibility were scaled across all cells in the pseudotime.

(I) Normalized ATAC signal across clusters identified in **(B)** within 100 kB around the *CEBPA* locus (chr19:33252563-33352564). The *CEBPA* +42kb cisRE is shown in orange. The *CEBPA* promoter is shown in purple. The four clusters taken for the pseudotime analysis were plotted separately on top and other clusters were sorted according to lineage. The heatmap on the right shows the aggregated scores for each lineage and MECOM-regulated cisRE insertions (identified in **Figure 4B-C**).

(J) Plots showing the normalized insertions in the *CEBPA* +42kb cisRE **(G)** (top left), lineage scores (top right), MECOM and CEBPA expression (bottom left) and MECOM and CEBPA TF motif enrichment (bottom right) across the pseudotime identified in **(A)** and **Figure 4**. Insertions, signature scores, expression and TF motif enrichment were scaled across all cells in the pseudotime and smoothened using LOESS.

Figure S6. Proof-of-concept gene editing strategy to inactivate the CEBPA cisRE with Cas9 nuclease

(A) Schematic showing gene editing strategy to inactivate *AAVS1*, *MECOM*, and *CEBPA* cisRE. (Left) *AAVS1* and *MECOM* were targeted with single sgRNAs targeting the early coding sequence (CDS). (Right) The *CEBPA* cisRE was targeted with two sgRNAs proximal to the summit of the MECOM ChIP-seq peak to create a 37bp inactivating deletion. In a proof-of-concept

experiment for this gene editing approach, WT MUTZ-3 cells were electroporated with Cas9 RNPs targeting *AAVS1* and *MECOM* with and without *CEBPA* cisRE targeting.

(B) Efficiency of gene editing in MUTZ-3 cells at the *AAVS1*, *MECOM*, and *CEBPA* (cisRE) loci. Editing estimated using Sanger sequencing of amplicons followed by sequence trace decomposition analysis with the ICE tool[13]. For *CEBPA* cisRE, only deletions resulting from dual guide cleavage were counted. n = 3 independent replicates, mean and SEM are shown.

(C-D) Immunophenotypic analysis of MUTZ-3 cells 4 days post-electroporation with Cas9 RNPs. **(C)** Histogram showing CD34 expression assessed by flow cytometry. **(D)** CD34 expression measured by MFI. n = 3 independent replicates, mean and SEM are shown. Two-sided Student t test was used for comparison. ****p < 0.0001. ns, not significant.

(E-F) CD34 expression measured by MFI from experiments on primary leukemia patient samples (patients 2-3, **Table S9**) in **Fig. 6.** n = 3 independent technical replicates, mean and SEM are shown. A Mann-Whitney Test was used for comparison. *p < 0.05, ****p < 0.0001.

(G) Viable cell counts by trypan blue exclusion in primary leukemia sample (patient 1, **Table S9**) 8 days post-electroporation from **Fig. 6.** n = 3 independent replicates, mean and SEM are shown. Two-sided Student t test was used for comparison. **p < 0.01, ns, not significant.

Figure S7. Activation of CEBPA cisRE induces myeloid differentiation of additional primary AML samples

(A-B) Immunophenotypic analysis of primary leukemia sample (patient 4, **Table S9**) 6 days post-electroporation with dCas9-VPR mRNA and sgRNAs targeting *CEBPA* cisRE or NT **(A)** Histograms showing CD34 and CD11b expression assessed by flow cytometry. **(B)** CD34 and CD11b expression measured by MFI. n = 3 independent replicates, mean and SEM are shown. Two-sided Student t test was used for comparison. **p < 0.01, ***p < 0.001.

(C-D) Immunophenotypic analysis of primary leukemia sample (patient 5, **Table S9**) 6-8 days post-electroporation with dCas9-VPR mRNA and sgRNAs targeting *CEBPA* cisRE or NT **(C)** Histograms showing CD34 (day 6) and CD11b (day 8) expression assessed by flow cytometry. **(D)** CD34 and CD11b expression measured by MFI. n = 3 independent replicates, mean and SEM are shown. Two-sided Student t test was used for comparison. ****p < 0.0001, *p < 0.05.

(E) Quantification of human cells in the bone marrow of mice transplanted with $1e^6$ primary AML cells. Viable cell counts were determined by trypan blue exclusion. Cell quantities were calculated based on total cell counts observed in the bone marrow and the corresponding human cell chimerism ($hCD45^+$). n = 4-10 xenotransplant recipients as shown, mean and SEM are shown. Two-sided Student t test was used for comparison. *p < 0.05.

(F) Quantification of leukemia initiating cell (LIC) frequencies based on bone marrow engraftment rates using the ELDA software[6]. Engraftment of leukemia cells was considered successful if human cell chimerism in the bone marrow was >5%.

(G-H) Weight (mg) and images of spleens from mice transplanted with $1e^6$ primary AML cells. n = 4-10 xenotransplant recipients as shown, mean and SEM are shown. Two-sided Student t test was used for comparison. *$p < 0.05$.

SUPPLEMENTARY TABLES

Supplementary Table 1. sgRNA sequences

Supplementary Table 2. RT-qPCR and genotyping primers

Supplementary Table 3. Bulk RNA-seq DMSO vs. dTAG^{V}-1 6- and 24-hour DGE analysis

Supplementary Table 4. PRO-seq DMSO vs dTAG^{V}-1 1- and 4-hour DGE analysis

Supplementary Table 5. ATAC-seq DMSO vs. dTAG^{V}-1 6-hour differential chromatin accessibility analysis

Supplementary Table 6. Consensus MECOM-regulated cisRE list with MECOM ChIP-seq overlap.

Supplementary Table 7. Consensus MECOM-regulated gene list

Supplementary Table 8. CRISPRi/a screen sgRNA enrichment quantification

Supplementary Table 9. Primary AML patient sample characteristics

Supplementary Table 10. Genomic mutations identified by WGS of AML samples analyzed in Figure 3

References

1. Bao, E. L. *et al.* Inherited myeloproliferative neoplasm risk affects haematopoietic stem cells. *Nature* **586**, 769–775 (2020).
2. Tomellini, E. *et al.* Integrin-α3 is a functional marker of ex vivo expanded human long-term hematopoietic stem cells. *Cell Rep.* **28**, 1063-1073.e5 (2019).
3. Lambo, S. *et al.* A longitudinal single-cell atlas of treatment response in pediatric AML. *Cancer Cell* **41**, 2117-2135.e12 (2023).
4. Kappas, N. C. & Bautch, V. L. Maintenance and in vitro differentiation of mouse embryonic stem cells to form blood vessels. *Curr. Protoc. Cell Biol.* **Chapter 23**, Unit 23.3 (2007).
5. McIntosh, B. E. *et al.* Nonirradiated NOD,B6.SCID Il2rγ-/- Kit(W41/W41) (NBSGW) mice support multilineage engraftment of human hematopoietic cells. *Stem Cell Reports* **4**, 171–180 (2015).
6. Hu, Y. & Smyth, G. K. ELDA: extreme limiting dilution analysis for comparing depleted and enriched populations in stem cell and other assays. *J. Immunol. Methods* **347**, 70–78 (2009).
7. Trombetta, J. J. *et al.* Preparation of single-cell RNA-Seq libraries for next generation sequencing. *Curr. Protoc. Mol. Biol.* **107**, 4.22.1-4.22.17 (2014).
8. Corces, M. R. *et al.* An improved ATAC-seq protocol reduces background and enables interrogation of frozen tissues. *Nat. Methods* **14**, 959–962 (2017).
9. Reimer, K. A., Mimoso, C. A., Adelman, K. & Neugebauer, K. M. Co-transcriptional splicing regulates 3’ end cleavage during mammalian erythropoiesis. *Mol. Cell* **81**, 998-1012.e7 (2021).
10. Bak, R. O., Dever, D. P. & Porteus, M. H. CRISPR/Cas9 genome editing in human hematopoietic stem cells. *Nat. Protoc.* **13**, 358–376 (2018).
11. Yamazaki, H. *et al.* A remote GATA2 hematopoietic enhancer drives leukemogenesis in

inv(3)(q21;q26) by activating EVI1 expression. *Cancer Cell* **25**, 415–427 (2014).

12. Gröschel, S. *et al.* A single oncogenic enhancer rearrangement causes concomitant EVI1 and GATA2 deregulation in leukemia. *Cell* **157**, 369–381 (2014).
13. Conant, D. *et al.* Inference of CRISPR edits from Sanger trace data. *CRISPR J.* **5**, 123–130 (2022).
14. Robert, M.-A. *et al.* Manufacturing of recombinant adeno-associated viruses using mammalian expression platforms. *Biotechnol. J.* **12**, 1600193 (2017).
15. Navarrete-Perea, J., Yu, Q., Gygi, S. P. & Paulo, J. A. Streamlined tandem mass tag (SL-TMT) protocol: An efficient strategy for quantitative (phospho)proteome profiling using tandem mass tag-synchronous precursor selection-MS3. *J. Proteome Res.* **17**, 2226–2236 (2018).
16. Yao, D. *et al.* Multicenter integrated analysis of noncoding CRISPRi screens. *Nat. Methods* **21**, 723–734 (2024).
17. Hill, A. J. *et al.* On the design of CRISPR-based single-cell molecular screens. *Nat. Methods* **15**, 271–274 (2018).
18. Choi, J. K. *et al.* Hybrid HIV/MSCV LTR enhances transgene expression of lentiviral vectors in human CD34(+) hematopoietic cells. *Stem Cells* **19**, 236–246 (2001).
19. Voit, R. A. *et al.* A genetic disorder reveals a hematopoietic stem cell regulatory network co-opted in leukemia. *Nat. Immunol.* **24**, 69–83 (2023).
20. Fulco, C. P. *et al.* Systematic mapping of functional enhancer-promoter connections with CRISPR interference. *Science* **354**, 769–773 (2016).
21. Najm, F. J. *et al.* Orthologous CRISPR-Cas9 enzymes for combinatorial genetic screens. *Nat. Biotechnol.* **36**, 179–189 (2018).
22. Jensen, T. I. *et al.* Targeted regulation of transcription in primary cells using CRISPRa and CRISPRi. *Genome Res.* **31**, 2120–2130 (2021).
23. Castro-Mondragon, J. A. *et al.* JASPAR 2022: the 9th release of the open-access database

of transcription factor binding profiles. *Nucleic Acids Res.* **50**, D165–D173 (2022).

24. Pastoors, D. *et al.* Oncogene EVI1 drives acute myeloid leukemia via a targetable interaction with CTBP2. *Sci. Adv.* **10**, eadk9076 (2024).
25. Fang, Z., Liu, X. & Peltz, G. GSEApy: a comprehensive package for performing gene set enrichment analysis in Python. *Bioinformatics* **39**, (2023).
26. Huttlin, E. L. *et al.* A tissue-specific atlas of mouse protein phosphorylation and expression. *Cell* **143**, 1174–1189 (2010).
27. Elias, J. E. & Gygi, S. P. Target-decoy search strategy for increased confidence in large-scale protein identifications by mass spectrometry. *Nat. Methods* **4**, 207–214 (2007).
28. Beausoleil, S. A., Villén, J., Gerber, S. A., Rush, J. & Gygi, S. P. A probability-based approach for high-throughput protein phosphorylation analysis and site localization. *Nat. Biotechnol.* **24**, 1285–1292 (2006).
29. McAlister, G. C. *et al.* Increasing the multiplexing capacity of TMTs using reporter ion isotopologues with isobaric masses. *Anal. Chem.* **84**, 7469–7478 (2012).
30. Li, W. *et al.* MAGeCK enables robust identification of essential genes from genome-scale CRISPR/Cas9 knockout screens. *Genome Biol.* **15**, 554 (2014).
31. Hao, Y. *et al.* Integrated analysis of multimodal single-cell data. *Cell* **184**, 3573-3587.e29 (2021).
32. Finak, G. *et al.* MAST: a flexible statistical framework for assessing transcriptional changes and characterizing heterogeneity in single-cell RNA sequencing data. *Genome Biol.* **16**, 278 (2015).
33. Hänzelmann, S., Castelo, R. & Guinney, J. GSVA: gene set variation analysis for microarray and RNA-seq data. *BMC Bioinformatics* **14**, 7 (2013).
34. Granja, J. M. *et al.* ArchR is a scalable software package for integrative single-cell chromatin accessibility analysis. *Nat. Genet.* **53**, 403–411 (2021).
35. van Dijk, D. *et al.* Recovering gene interactions from single-cell data using data diffusion.

Cell **174**, 716-729.e27 (2018).

36. Schep, A. N., Wu, B., Buenrostro, J. D. & Greenleaf, W. J. chromVAR: inferring transcription-factor-associated accessibility from single-cell epigenomic data. *Nat. Methods* **14**, 975–978 (2017).
37. Weirauch, M. T. *et al.* Determination and inference of eukaryotic transcription factor sequence specificity. *Cell* **158**, 1431–1443 (2014).
38. Trapnell, C. *et al.* The dynamics and regulators of cell fate decisions are revealed by pseudotemporal ordering of single cells. *Nat. Biotechnol.* **32**, 381–386 (2014).
39. Aplenc, R. *et al.* Bortezomib with standard chemotherapy for children with acute myeloid leukemia does not improve treatment outcomes: a report from the Children's Oncology Group. *Haematologica* **105**, 1879–1886 (2020).

Figure S1

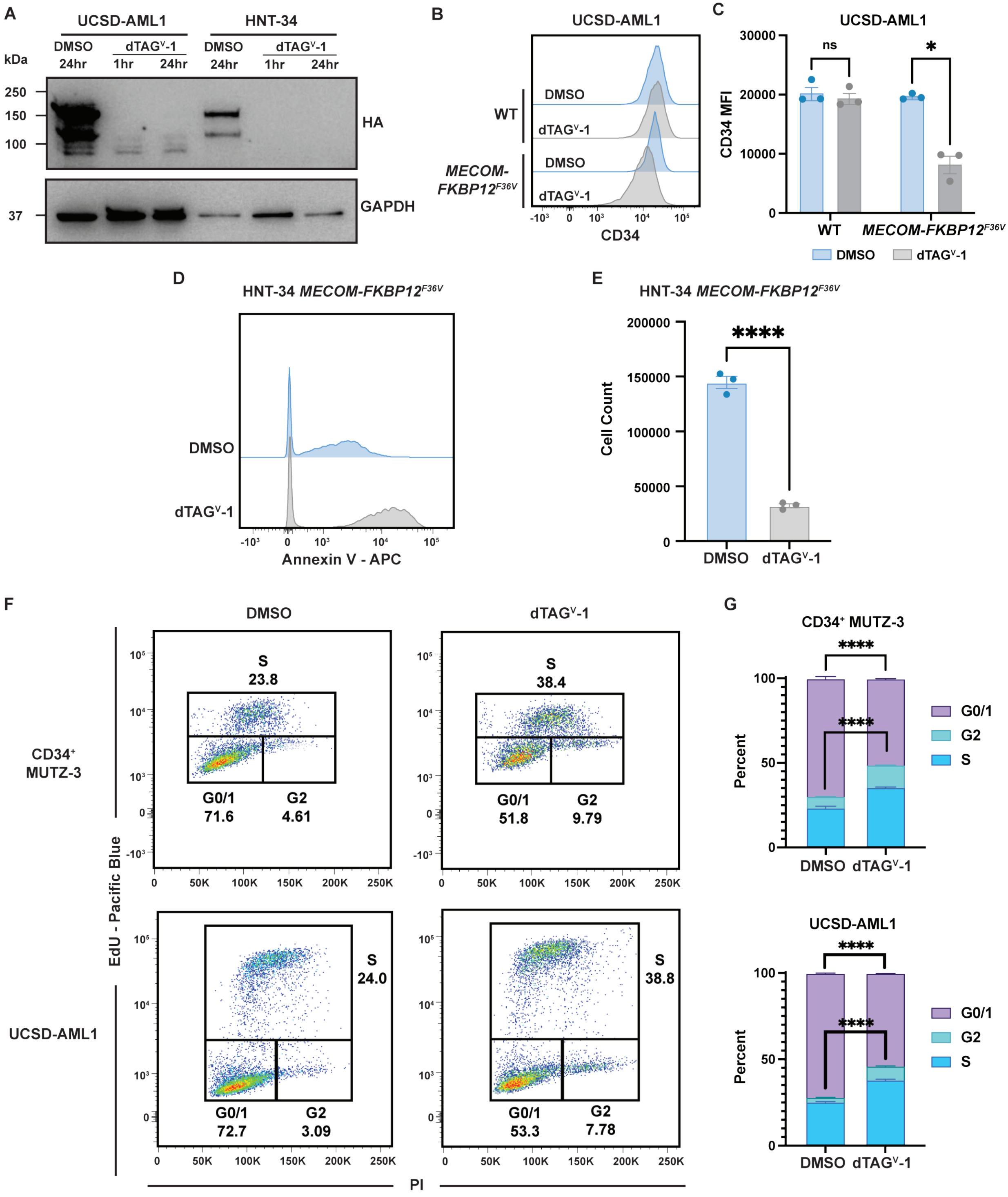

A
UCSD-AML1
HNT-34
DMSO 24hr
dTAG^V-1 1hr 24hr
kDa
250
150
100
37
HA
GAPDH
B
UCSD-AML1
WT
MECOM-FKBP12^F36V
DMSO
dTAG^V-1
CD34
C
UCSD-AML1
CD34 MFI
ns
*
WT
MECOM-FKBP12^F36V
DMSO
dTAG^V-1
D
HNT-34 MECOM-FKBP12^F36V
Annexin V - APC
E
HNT-34 MECOM-FKBP12^F36V
Cell Count
****
F
CD34+ MUTZ-3
UCSD-AML1
EdU - Pacific Blue
PI
S 23.8
G0/1 71.6
G2 4.61
S 38.4
G0/1 51.8
G2 9.79
S 24.0
G0/1 72.7
G2 3.09
S 38.8
G0/1 53.3
G2 7.78
G
CD34+ MUTZ-3
UCSD-AML1
Percent
G0/1
G2
S

Figure S2

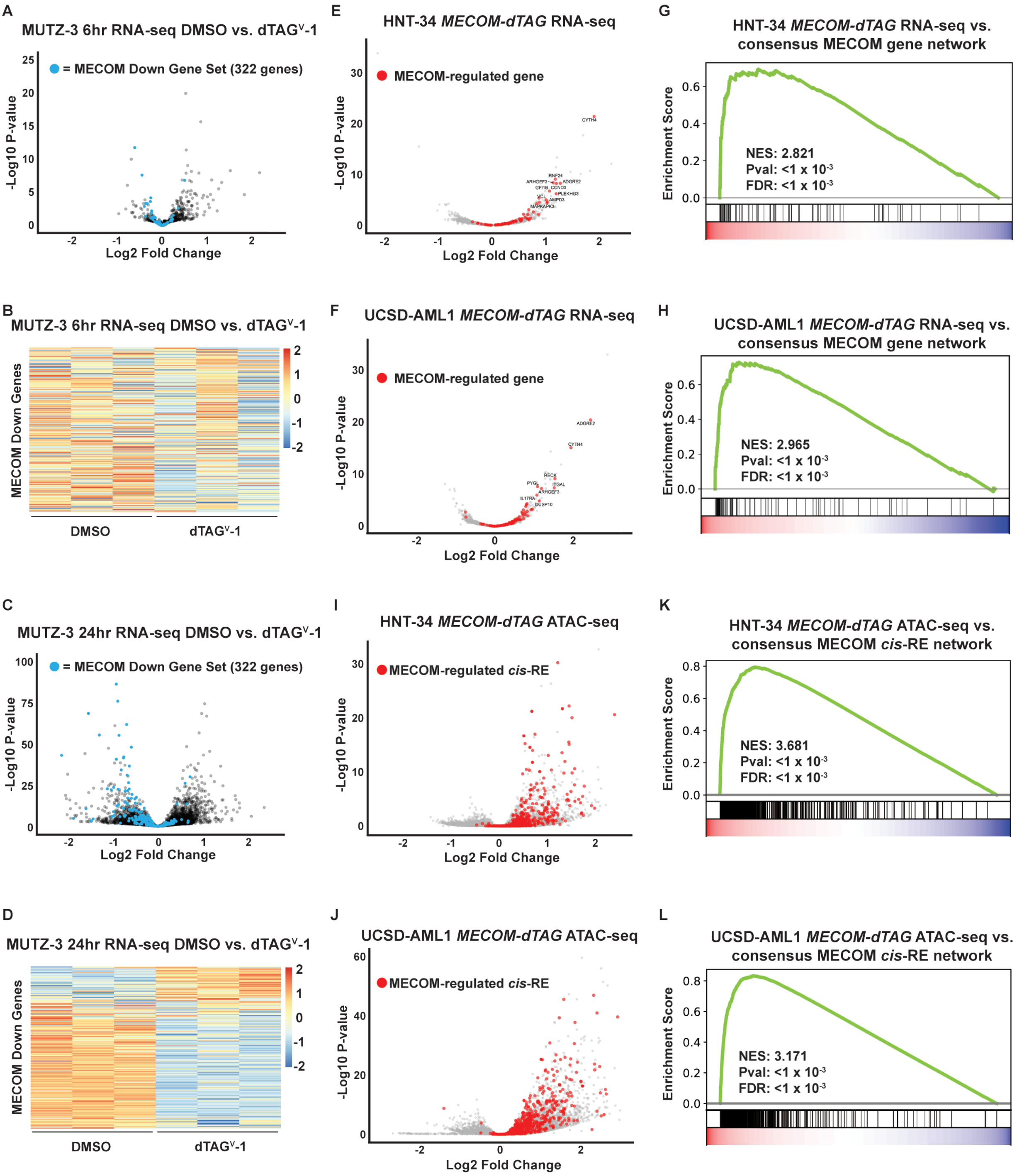
A
MUTZ-3 6hr RNA-seq DMSO vs. dTAG^{V}-1
= MECOM Down Gene Set (322 genes)
-Log10 P-value
Log2 Fold Change
B
MUTZ-3 6hr RNA-seq DMSO vs. dTAG^{V}-1
MECOM Down Genes
DMSO
dTAG^{V}-1
C
MUTZ-3 24hr RNA-seq DMSO vs. dTAG^{V}-1
= MECOM Down Gene Set (322 genes)
-Log10 P-value
Log2 Fold Change
D
MUTZ-3 24hr RNA-seq DMSO vs. dTAG^{V}-1
MECOM Down Genes
DMSO
dTAG^{V}-1
E
HNT-34 *MECOM-dTAG* RNA-seq
MECOM-regulated gene
CYTH4
RNF24
ARHGEF3
ADGRE2
GFI1B
CCND3
PLEKHG3
VCL
AMPD3
MAPKAPK3
-Log10 P-value
Log2 Fold Change
F
UCSD-AML1 *MECOM-dTAG* RNA-seq
MECOM-regulated gene
ADGRE2
CYTH4
RECK
PYGL
ITGAL
ARHGEF3
IL17RA
DUSP10
-Log10 P-value
Log2 Fold Change
G
HNT-34 *MECOM-dTAG* RNA-seq vs. consensus MECOM gene network
Enrichment Score
NES: 2.821
Pval: <1 x 10^{-3}
FDR: <1 x 10^{-3}
H
UCSD-AML1 *MECOM-dTAG* RNA-seq vs. consensus MECOM gene network
Enrichment Score
NES: 2.965
Pval: <1 x 10^{-3}
FDR: <1 x 10^{-3}
I
HNT-34 *MECOM-dTAG* ATAC-seq
MECOM-regulated *cis*-RE
-Log10 P-value
Log2 Fold Change
J
UCSD-AML1 *MECOM-dTAG* ATAC-seq
MECOM-regulated *cis*-RE
-Log10 P-value
Log2 Fold Change
K
HNT-34 *MECOM-dTAG* ATAC-seq vs. consensus MECOM *cis*-RE network
Enrichment Score
NES: 3.681
Pval: <1 x 10^{-3}
FDR: <1 x 10^{-3}
L
UCSD-AML1 *MECOM-dTAG* ATAC-seq vs. consensus MECOM *cis*-RE network
Enrichment Score
NES: 3.171
Pval: <1 x 10^{-3}
FDR: <1 x 10^{-3}

Figure S3

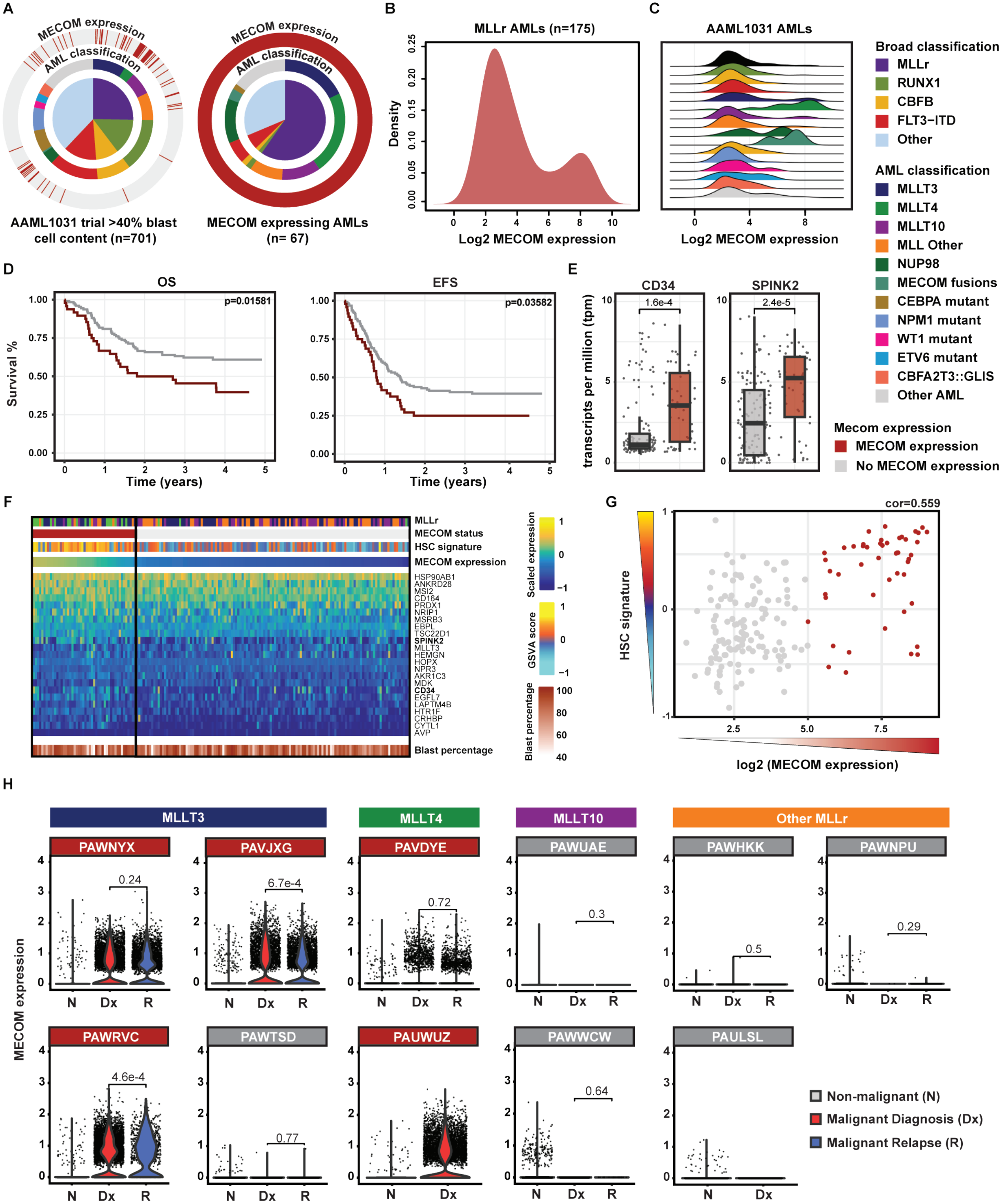

A
MECOM expression
AML classification
AAML1031 trial >40% blast cell content (n=701)
MECOM expressing AMLs (n= 67)
B
MLLr AMLs (n=175)
Density
Log2 MECOM expression
C
AAML1031 AMLs
Log2 MECOM expression
Broad classification
MLLr
RUNX1
CBFB
FLT3−ITD
Other
AML classification
MLLT3
MLLT4
MLLT10
MLL Other
NUP98
MECOM fusions
CEBPA mutant
NPM1 mutant
WT1 mutant
ETV6 mutant
CBFA2T3::GLIS
Other AML
D
OS
p=0.01581
EFS
p=0.03582
Survival %
Time (years)
E
CD34
1.6e-4
SPINK2
2.4e-5
transcripts per million (tpm)
Mecom expression
MECOM expression
No MECOM expression
F
MLLr
MECOM status
HSC signature
MECOM expression
HSP90AB1
ANKRD28
MSI2
CD164
PRDX1
NRIP1
MSRB3
EBPL
TSC22D1
SPINK2
MLLT3
HEMGN
NPR3
AKR1C3
MDK
CD34
EGFL7
LAPTM4B
HTR1F
CRHBP
CYTL1
AVP
Blast percentage
Scaled expression
GSVA score
Blast percentage
G
cor=0.559
HSC signature
log2 (MECOM expression)
H
MLLT3
MLLT4
MLLT10
Other MLLr
PAWNYX
PAVJXG
PAVDYE
PAWUAE
PAWHKK
PAWNPU
PAWRVC
PAWTSD
PAUWUZ
PAWWCW
PAULSL
0.24
6.7e-4
0.72
0.3
0.5
0.29
4.6e-4
0.77
0.64
MECOM expression
N
Dx
R
Non-malignant (N)
Malignant Diagnosis (Dx)
Malignant Relapse (R)

Figure S4

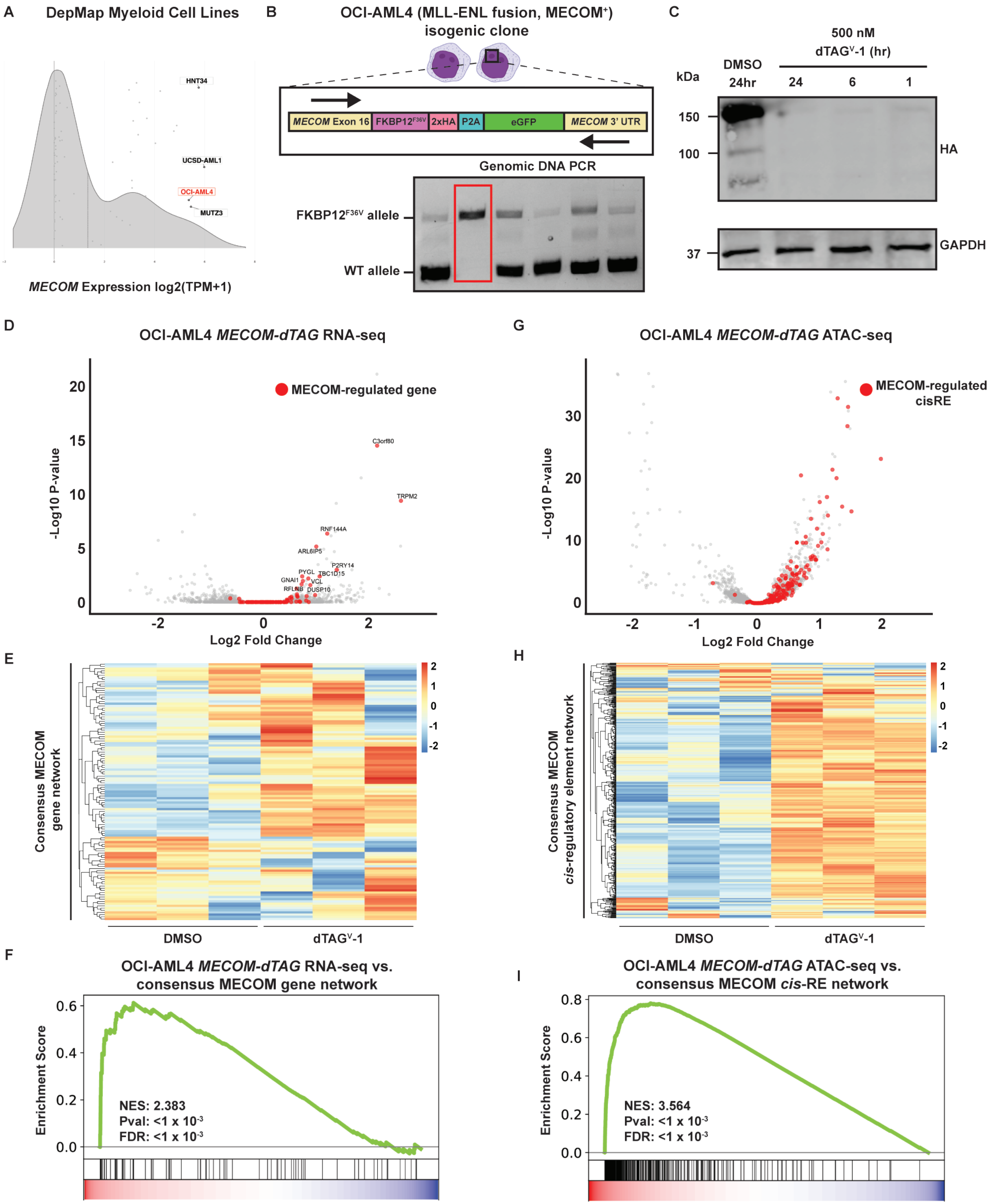

A
DepMap Myeloid Cell Lines
HNT34
UCSD-AML1
OCI-AML4
MUTZ3
MECOM Expression log2(TPM+1)
B
OCI-AML4 (MLL-ENL fusion, MECOM+) isogenic clone
MECOM Exon 16
FKBP12F36V
2xHA
P2A
eGFP
MECOM 3' UTR
Genomic DNA PCR
FKBP12F36V allele
WT allele
C
500 nM dTAGV-1 (hr)
kDa
DMSO 24hr
24
6
1
150
100
HA
37
GAPDH
D
OCI-AML4 MECOM-dTAG RNA-seq
MECOM-regulated gene
-Log10 P-value
Log2 Fold Change
C3orf80
TRPM2
RNF144A
ARL6IP5
P2RY14
PYGL
TBC1D15
GNAI1
VCL
RFLNB
DUSP10
G
OCI-AML4 MECOM-dTAG ATAC-seq
MECOM-regulated cisRE
-Log10 P-value
Log2 Fold Change
E
Consensus MECOM gene network
DMSO
dTAGV-1
H
Consensus MECOM cis-regulatory element network
DMSO
dTAGV-1
F
OCI-AML4 MECOM-dTAG RNA-seq vs. consensus MECOM gene network
Enrichment Score
NES: 2.383
Pval: <1 x 10-3
FDR: <1 x 10-3
I
OCI-AML4 MECOM-dTAG ATAC-seq vs. consensus MECOM cis-RE network
Enrichment Score
NES: 3.564
Pval: <1 x 10-3
FDR: <1 x 10-3

Figure S5

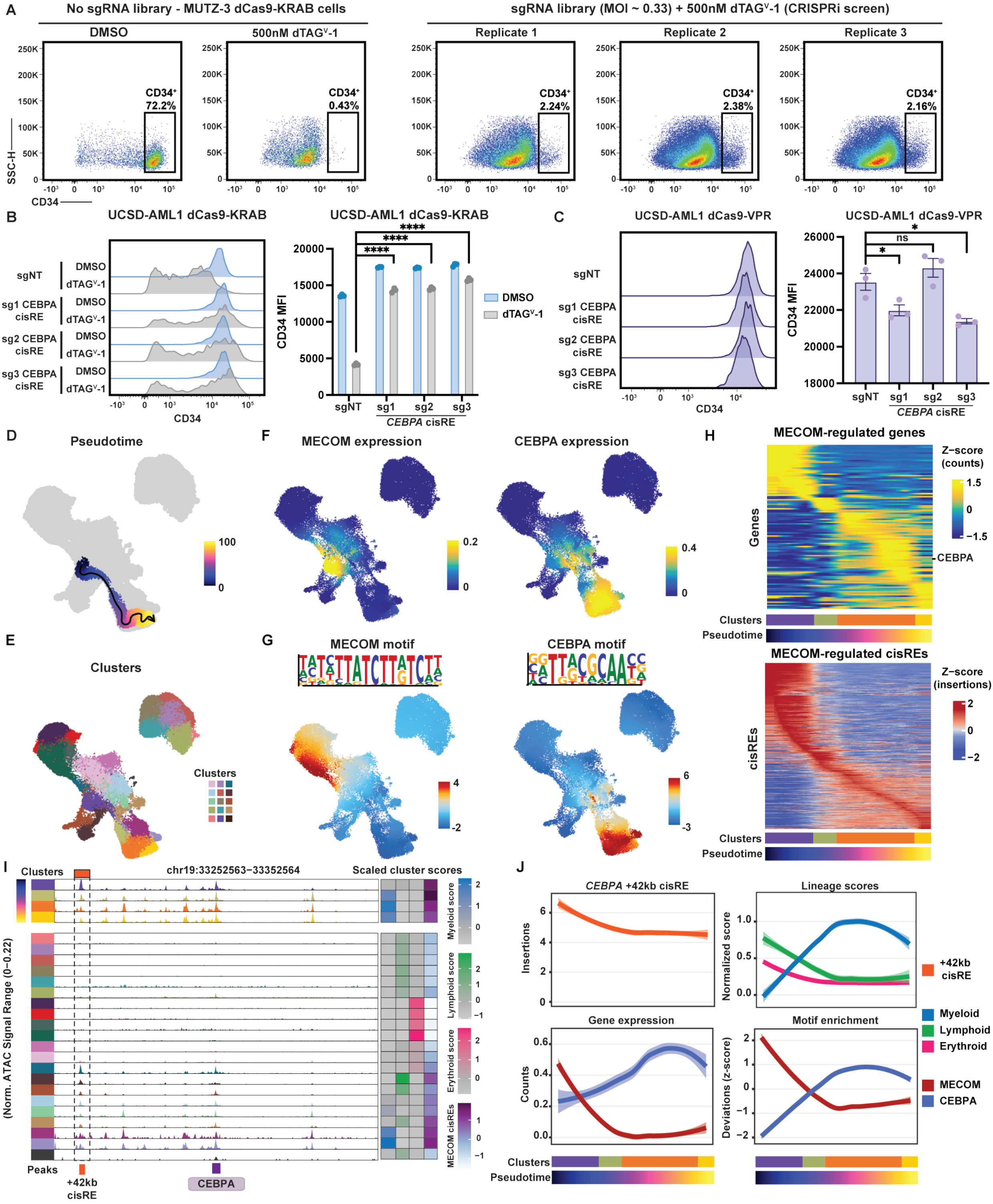

A
No sgRNA library - MUTZ-3 dCas9-KRAB cells
DMSO
500nM dTAGV-1
sgRNA library (MOI ~ 0.33) + 500nM dTAGV-1 (CRISPRi screen)
Replicate 1
Replicate 2
Replicate 3
CD34+ 72.2%
CD34+ 0.43%
CD34+ 2.24%
CD34+ 2.38%
CD34+ 2.16%
SSC-H
CD34
B
UCSD-AML1 dCas9-KRAB
sgNT
sg1 CEBPA cisRE
sg2 CEBPA cisRE
sg3 CEBPA cisRE
DMSO
dTAGV-1
CD34 MFI
CEBPA cisRE
C
UCSD-AML1 dCas9-VPR
ns
D
Pseudotime
E
Clusters
F
MECOM expression
CEBPA expression
G
MECOM motif
CEBPA motif
H
MECOM-regulated genes
Z-score (counts)
Genes
CEBPA
Pseudotime
MECOM-regulated cisREs
Z-score (insertions)
cisREs
I
chr19:33252563-33352564
Scaled cluster scores
Myeloid score
Lymphoid score
Erythroid score
MECOM cisREs
(Norm. ATAC Signal Range (0-0.22))
Peaks
+42kb cisRE
CEBPA
J
CEBPA +42kb cisRE
Insertions
Lineage scores
Normalized score
Gene expression
Counts
Motif enrichment
Deviations (z-score)
+42kb cisRE
Myeloid
Lymphoid
Erythroid
MECOM
CEBPA

Figure S6

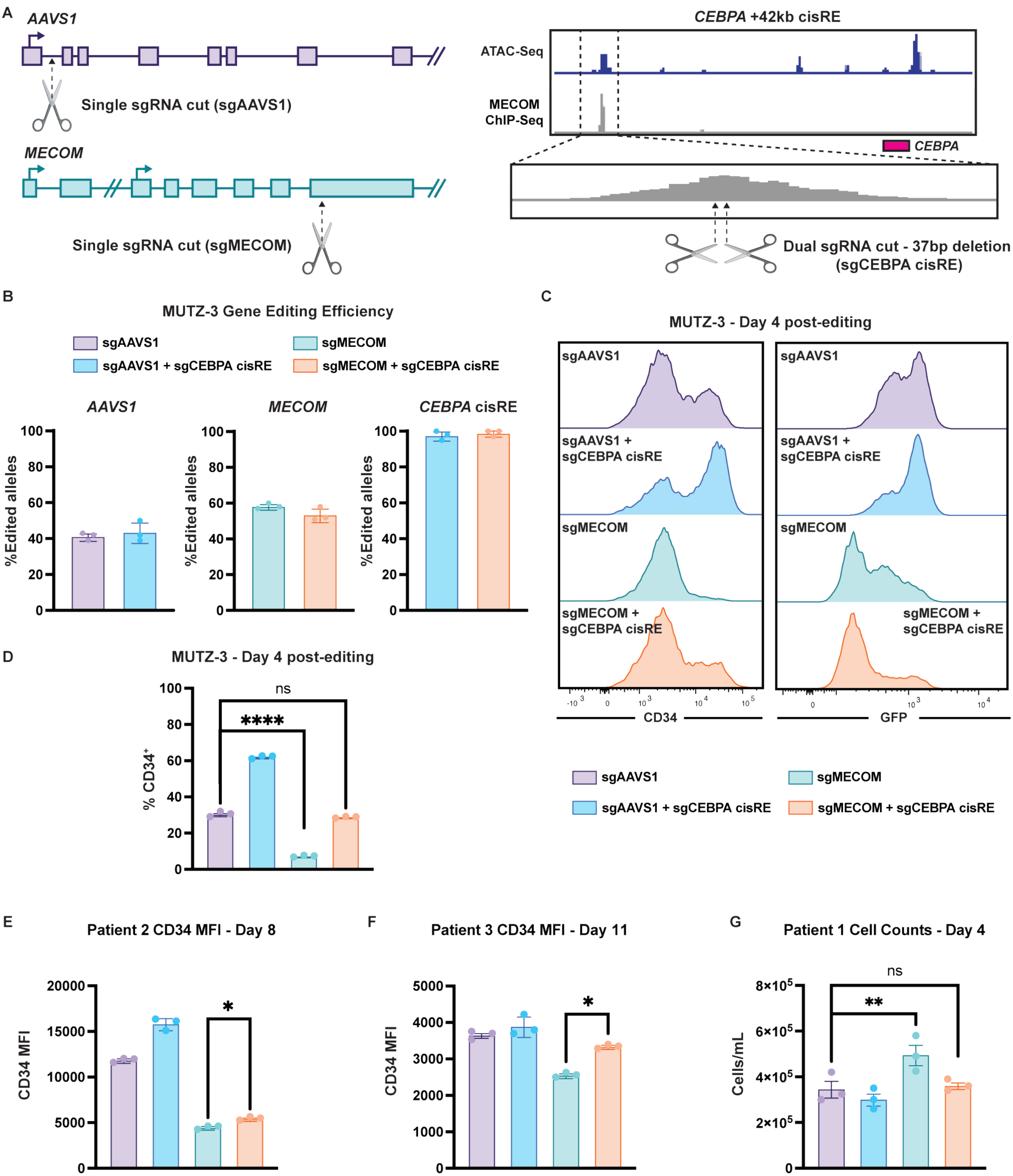
A
AAVS1
Single sgRNA cut (sgAAVS1)
MECOM
Single sgRNA cut (sgMECOM)
CEBPA +42kb cisRE
ATAC-Seq
MECOM ChIP-Seq
CEBPA
Dual sgRNA cut - 37bp deletion (sgCEBPA cisRE)
B
MUTZ-3 Gene Editing Efficiency
sgAAVS1
sgMECOM
sgAAVS1 + sgCEBPA cisRE
sgMECOM + sgCEBPA cisRE
AAVS1
MECOM
CEBPA cisRE
%Edited alleles
C
MUTZ-3 - Day 4 post-editing
sgAAVS1
sgAAVS1 + sgCEBPA cisRE
sgMECOM
sgMECOM + sgCEBPA cisRE
CD34
GFP
D
MUTZ-3 - Day 4 post-editing
ns
****
% CD34+
E
Patient 2 CD34 MFI - Day 8
CD34 MFI
*
F
Patient 3 CD34 MFI - Day 11
CD34 MFI
*
G
Patient 1 Cell Counts - Day 4
ns
**
Cells/mL

Figure S7

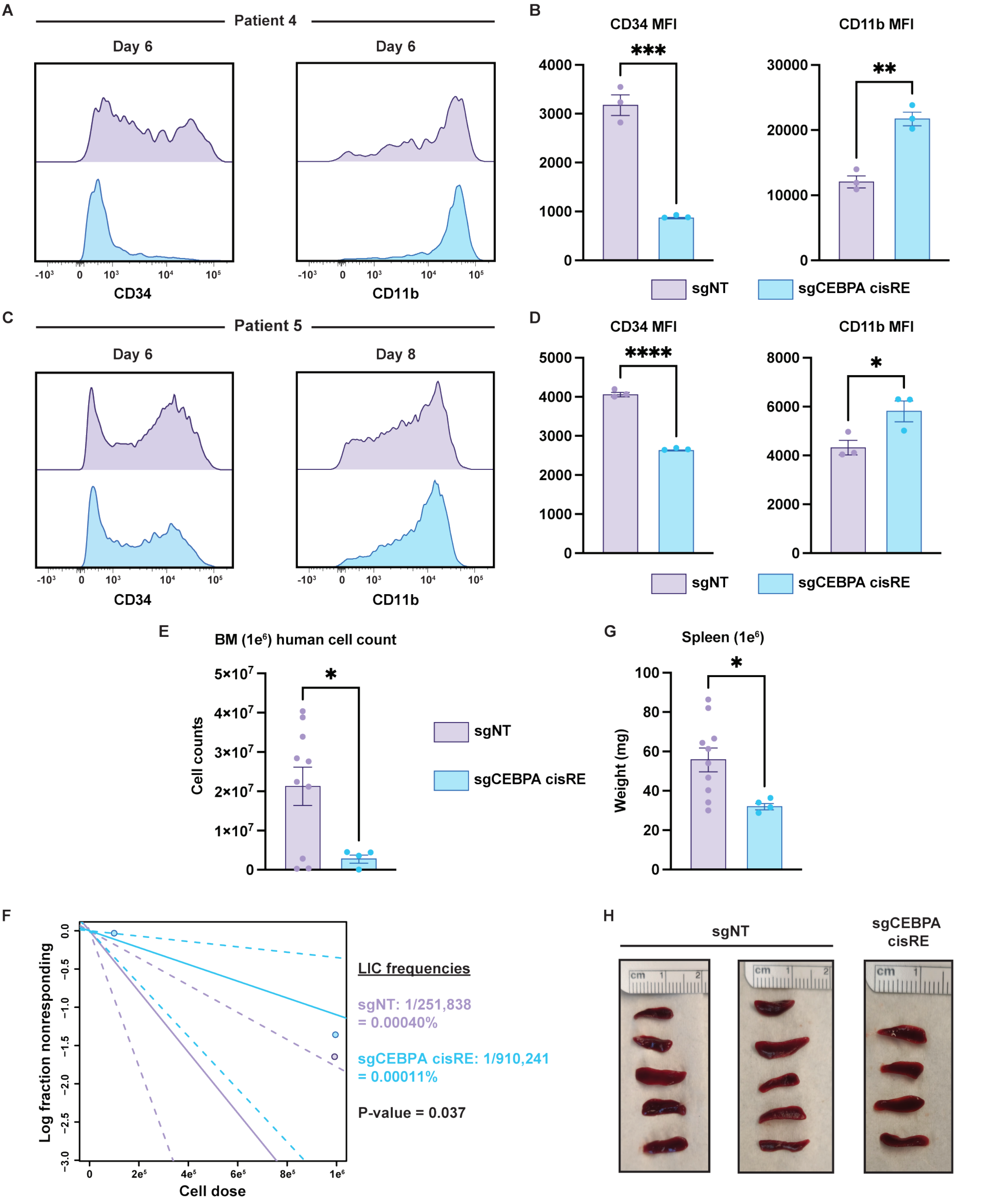
A
Patient 4
Day 6
Day 6
CD34
CD11b
B
CD34 MFI
CD11b MFI
***
**
sgNT
sgCEBPA cisRE
C
Patient 5
Day 6
Day 8
CD34
CD11b
D
CD34 MFI
CD11b MFI
****
*
sgNT
sgCEBPA cisRE
E
BM (1e6) human cell count
Cell counts
sgNT
sgCEBPA cisRE
G
Spleen (1e6)
Weight (mg)
F
Log fraction nonresponding
Cell dose
LIC frequencies
sgNT: 1/251,838
= 0.00040%
sgCEBPA cisRE: 1/910,241
= 0.00011%
P-value = 0.037
H
sgNT
sgCEBPA
cisRE
